# RetroBioCat Database: A Platform for Collaborative Curation and Automated Meta-Analysis of Biocatalysis Data

**DOI:** 10.1021/acscatal.3c01418

**Published:** 2023-08-22

**Authors:** William Finnigan, Max Lubberink, Lorna J. Hepworth, Joan Citoler, Ashley P. Mattey, Grayson J. Ford, Jack Sangster, Sebastian C. Cosgrove, Bruna Zucoloto da Costa, Rachel S. Heath, Thomas W. Thorpe, Yuqi Yu, Sabine L. Flitsch, Nicholas J. Turner

**Affiliations:** Department of Chemistry, Manchester Institute of Biotechnology, University of Manchester, 131 Princess Street, Manchester M1 7DN, U.K.

**Keywords:** biocatalysis, database, enzyme selection, visualization, synthesis planning

## Abstract

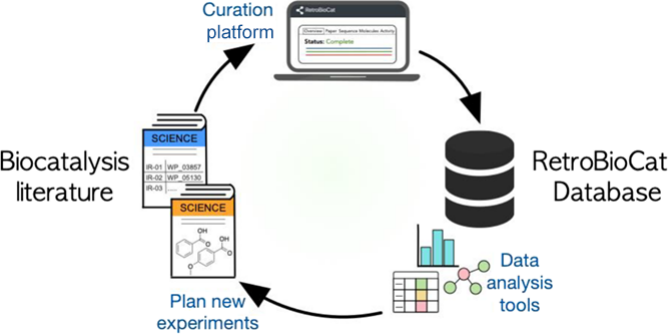

Despite the increasing use of biocatalysis for organic synthesis, there are currently no databases that adequately capture synthetic biotransformations. The lack of a biocatalysis database prevents accelerating biocatalyst characterization efforts from being leveraged to quickly identify candidate enzymes for reactions or cascades, slowing their development. The RetroBioCat Database (available at retrobiocat.com) addresses this gap by capturing information on synthetic biotransformations and providing an analysis platform that allows biocatalysis data to be searched and explored through a range of highly interactive data visualization tools. This database makes it simple to explore available enzymes, their substrate scopes, and how characterized enzymes are related to each other and the wider sequence space. Data entry is facilitated through an openly accessible curation platform, featuring automated tools to accelerate the process. The RetroBioCat Database democratizes biocatalysis knowledge and has the potential to accelerate biocatalytic reaction development, making it a valuable resource for the community.

## Introduction

Accelerating enzyme discovery and engineering efforts have created an increasingly broad palette of available biocatalytic reactions for chemists to work with.^1^ Concurrently, a drive for greener processes means biocatalysis is the method of choice for reactions that would be environmentally unfriendly or challenging to perform otherwise.^2^ However, understanding around which enzymes can be reliably used for organic synthesis, and what their substrate scope is, remains mostly in the hands of domain experts. Indeed, the risks and unknowns for whether a given biocatalytic reaction is feasible pose a hurdle for the uptake of biocatalysis.^3,4^ Better and more widely accessible biocatalyst informatics is key for tackling this bottleneck.

In addition, we are beginning to see an explosion in biocatalyst characterization data, in some cases with hundreds of enzymes reported in a single publication.^5^ Combining and analyzing the data from these publications is essential to understand the limitations and frontiers for an enzyme class. Such meta-analyses are often presented in the form of a review article, highlighting the reported activities across all the enzymes characterized and how sequences relate to each other. Tables of substrate scope, phylogenetic trees, sequence similarity networks (SSNs), and other visualizations are often manually crafted and presented.^6−10^ Review articles like these commonly act as an atlas for a scientist considering an enzyme class for the first time. However, as new enzymes are characterized, reviews can quickly become out of date, leaving the painstakingly compiled information stranded in a paper-based format.

**Figure 1:**
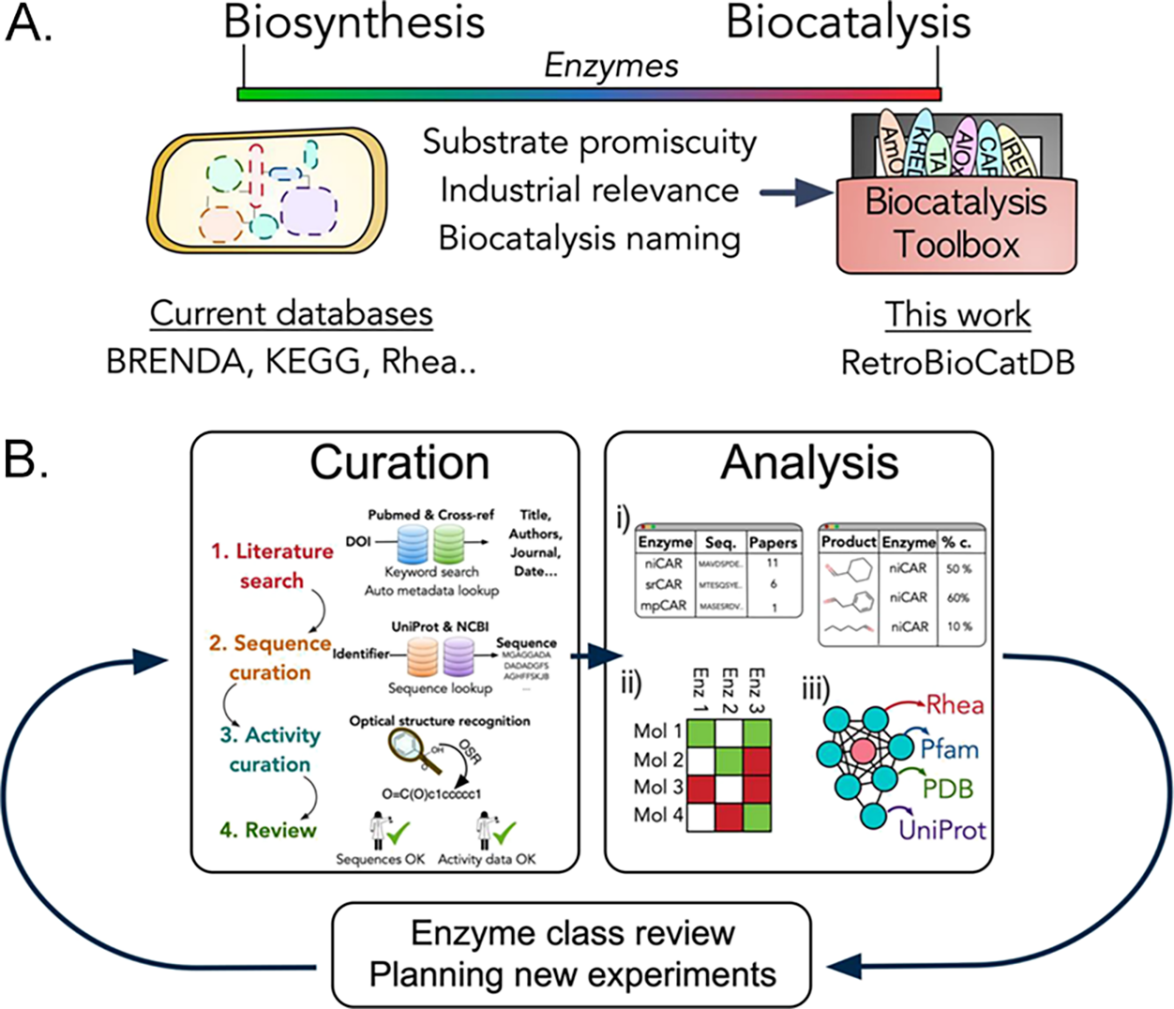
(A) Classification of enzymes in the biocatalysis toolbox often diverges from the entries in biosynthesis databases. Importantly, enzymes make their way into the biocatalysis toolbox by demonstrating substrate promiscuity and industrial relevance. (B) RetroBioCat-DB consists of both a curation platform and an analysis platform. The curation platform leverages tools such as optical structure recognition (OSR) queries to existing databases for rapid data entry. The analysis platform includes an array of tools and tables to explore biocatalysis data, including (i) queryable look-up tables for papers, sequences, or activity data, (ii) interactive heatmap diagrams showing which enzymes are active against which substrates, and (iii) interactive SSNs, with links to Rhea, Pfam, PDB, and UniProt, where these are available for homologous sequences. Data curated and analyzed in RetroBioCat-DB can be used to review an enzyme class or to help plan new experiments.

Publicly accessible databases offer a route to ensure that the body of knowledge for enzymes is continually updated. Indeed, biology databases such as BRENDA, Rhea, KEGG, and UniProt offer essential services in capturing the function of enzymes.^11−14^ However, reactions with synthetic substrates are often missing from these databases. Furthermore, the naming utilized in the biocatalysis literature often does not align with annotations in biology databases (Figure 1A). Some synthetic enzyme reactions are captured in commercial chemical databases such as Reaxys or SciFinder, yet enzyme sequence data and negative datapoints are missing, and many biocatalysis papers are not present. Importantly, existing databases do not offer the capability for a meta-analysis of what the synthetic substrate scope for an enzyme class is, what the best enzymes are, or how enzymes are related to each other and the wider sequence space. An overview of available biology and chemistry databases is available in Supplementary Table 1.

As highlighted elsewhere,^15^ a database specifically for biocatalysis is essential to make intelligent use of accelerating biocatalyst characterization efforts. Here, we present the RetroBioCat Database (RetroBioCat-DB), an integrated platform for curating and analyzing biocatalysis data (Figure 1B). The platform is available at retrobiocat.com and provides a set of interactive tools to explore the available biocatalysis enzymes, their substrate scope, and how these enzymes are related to both each other and uncharacterized enzymes.

## Materials and Methods

### Overview

RetroBioCat-DB uses a python Flask web server that employs Jinja2 to render HTML pages, utilizing Bootstrap 4 and custom Javascript to provide the user interface. To create data visualizations, the web application employs various Javascript libraries including Tabulator, BokehJS, and VisJS. Bokeh graphs are generated server-side in python as required. The RDKit python library is used to implement all chemistry-related methods. Additionally, the Scikit-learn, Pandas, Biopython, and NumPy python packages are employed as required. MongoDB is used to store all data, primarily accessed via the MongoEngine python package. Data to create visualizations and tables is accessed using MongoEngine queries. Further details on the database structure and the different activity data types captured are available in the Supporting Information (Supplementary Figure 1).

### Specific Tools

#### Sequence Similarity Networks

For each new protein sequence entered into the database, automated BLAST searches are carried out using the EBI REST API,^16^ against the UniRef50 database. Sequences with more than 80% coverage, more than 30% sequence identity, and less than 20% larger or smaller than the query sequence are retained. Further information including Pfam domains and any associated Rhea reactions is retrieved from UniProt. Once all UniRef50 homologues are retrieved, automated all-vs-all BLAST searches are carried out for every RetroBioCat-DB and UniRef50 sequence, and an alignment score is calculated for every pair of sequences.^17^ SSNs are then created using these alignments, with network positioning precomputed at a range of alignment scores to allow rapid visualization. A representation of this workflow is shown in Supplementary Figure 2.

#### Heatmaps

Data from which to create a heatmap is accessed using a MongoEngine query. From this data, lists of unique enzymes and unique molecules are identified. A dendrogram of molecules is created using agglomerative clustering with Tanimoto similarity used as the distance metric. A dendrogram of sequences is also created, using the Euclidean distance between UniRep embeddings as the distance metric. A heatmap is then generated using Bokeh, visualizing activity for every enzyme–substrate pair where there is data. The heatmap includes custom Javascript callbacks to allow further data exploration as required.

#### Substrate Summary Tool

The substrate summary tool is created using a modified version of EHReact.^18^ Seed molecules are predefined for each reaction and used to create a Hasse diagram given a set of unique molecules for a reaction. Initially, the first step in the Hasse diagram is shown, with the user being able to either select a particular branch to explore further or simply see all the molecules in a branch.

#### Enzyme and Product Similarity Searches

The enzyme sequences in RetroBioCat-DB can be queried using the NCBI BLAST tool,^19^ with a minimum *E*-value of 0.5 and maximum alignments set to 1000. For each high-scoring pair, coverage is calculated as the alignment length over the query length and identity as identities over the alignment length. An alignment score is also calculated, as reported elsewhere.^17^ Product similarity searches are carried out using precomputed RDKit fingerprints for every molecule in RetroBioCat-DB, which are used to score Tanimoto similarity to a query molecule, with results above a cut-off value returned. Where multiple entries are available for a similar product molecule, these are ranked first by their categorical activity value (high, medium, or low) and then again by any specific activity or conversion values, with specific activity values favored over conversion. Only the best entry per enzyme is kept before the top n ranked activities are returned for each product.

## Results and Discussion

### Database Scope and Design

We centered RetroBioCat-DB around enzyme classes as they are commonly described in the biocatalysis literature, constituting what is commonly thought of as the enzyme toolbox for biocatalysis (Figure 1A). Many reviews describe these enzymes, which differentiate themselves from other enzymes by having become established as useful for synthetic reactions in organic chemistry.^2,15,20,21^ Indeed, these are the enzymes suggested during synthesis planning by RetroBioCat.^22^

Importantly, the naming of enzymes in biocatalysis can differ from annotations in biology databases (Figure 1A). Enzymes in biocatalysis are often named after the broad type of chemistry they can catalyze rather than any specific metabolic function or structural fold. For example, imine reductase (IRED), an enzyme that has found numerous uses in industrial biocatalysis,^23,24^ has no bespoke entry in BRENDA, Rhea, or KEGG.^11,12,14^ Indeed, the naming of biocatalysis enzymes often reflects the goal that these enzymes can be thought of simply as catalysts which are able to work robustly on multiple substrates.^3^

### Data Curation

#### Building a Highly Accessible Data Curation Platform

**Figure 2:**
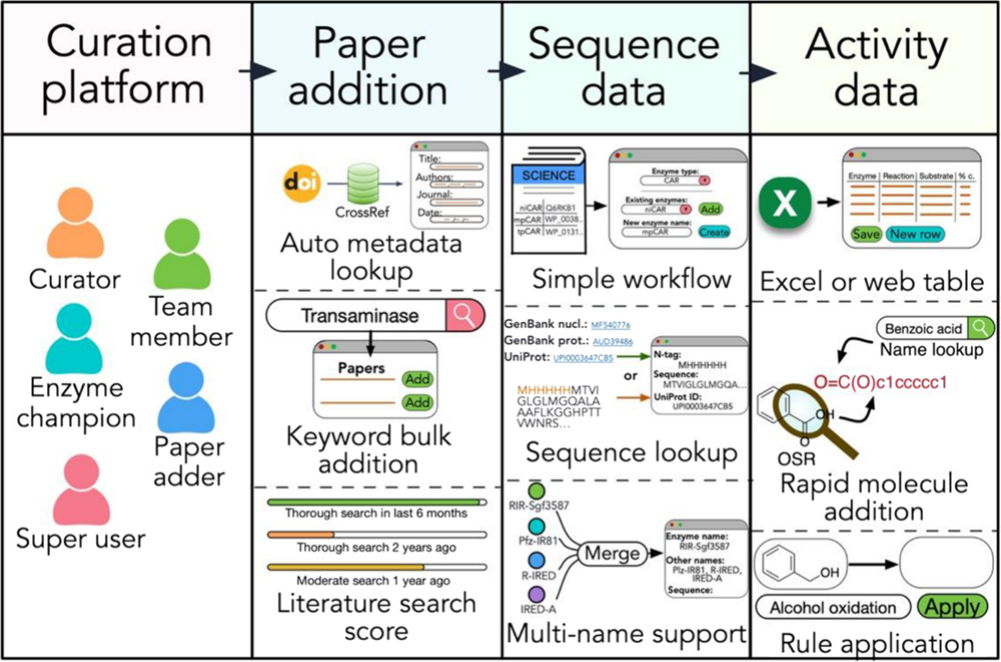
Curation platform: users can be assigned roles with differing levels of access and the ability to mark curated data as reviewed. Paper addition: papers for curation can be added either by specifying a DOI, from which metadata is automatically retrieved, or in bulk by using keyword searches to PubMed. To capture the database coverage for a given enzyme class, a literature search score can be entered by a user, which will decrease over time. Sequence data: for each paper, sequences can be added using a simple web portal, which allows existing sequence entries to be re-used or new sequences to be created. UniProt or GenBank identifiers can be utilized to automatically download relevant amino acid sequences. Entries with identical amino acid sequences can later be merged into a single entity to facilitate data analysis. Activity data: activity data can be added using a web portal or uploaded as an excel file. OSR can be utilized to rapidly add molecules, which can be named and used in the place of SMILES strings when recording activity. Reaction rules can also be applied to the molecules entered for the creation of the corresponding products or substrates.

Despite the promise of automated reaction extraction and text mining, the generation of high-quality structured datasets still relies mostly on manual curation. Indeed, manual curation is standard across biological databases.^11−14^ A recent example is the Natural Products Atlas,^25^ which utilizes a crowd-sourcing approach for data entry through a web portal. Similarly, ProtaBank also utilizes crowd-sourced data entry for protein engineering data.^26^ Inspired by these approaches, we sought to create an openly accessible web portal for the curation of biocatalysis data, with tools to augment the curation process where possible. New data can be added to RetroBioCat-DB by anyone, with varying levels of access and a review process to ensure that only high-quality data is incorporated into the database (Figure 2). Importantly, data is attributed to the user who added it.

Data entry is organized by paper. Users with suitable access can add papers either directly via their DOI or through an interface for bulk paper addition using keyword searches to PubMed. Metadata is automatically added from CrossRef or PubMed, although this can be manually altered if necessary. A user-entered score for how thorough the literature has been searched can also be recorded (Supplementary Figures 3–5). Users can assign themselves papers for curation, launching the data submission portal consisting of four tabs: status, paper, sequence, and activity. The sequence and activity tabs are for curating the data recorded in the paper, consisting of the enzyme amino acid sequences and associated information and the activity these enzymes have shown. Semi-automated tools are embedded into this workflow to augment the curation process (Figure 2). Examples of the curation platform are shown in the Supporting Information (Supplementary Figures 6–16).

#### Enzyme Naming Differences and Sequence Tags

In many cases, identical enzymes are reported with different names, making comparisons across publications challenging. Even in the cases where these differences are acknowledged, identical numbering schemes can create a confusing situation.^27^ To tackle this challenge, we incorporated a workflow to identify identical amino acid sequences, with the option to merge entries under a single given name where sequences match (Figure 2 and Supplementary Figure 16). Where merging occurs, the additional names are saved along with any alternative sequence tags or notes. Alternative naming can also be set for use during activity data curation.

An additional challenge when comparing sequences is the addition of N or C terminal tags, which are commonly used to aid in protein purification. Enzymes which are otherwise identical can be reported with differences in the tag used. RetroBioCat-DB handles terminal tags by recording them separately from the main amino acid sequence (Figure 2 and Supplementary Figure 10). While this approach has some drawbacks, recording tags separately facilitates comparison of work published on otherwise identical proteins and simpler connections to existing protein databases.

#### Achieving Database Completion

Complete coverage of all biocatalysis data is a significant undertaking and likely will be an ongoing challenge for RetroBioCat-DB. However, each enzyme type acts as its own mini database, offering insight into what sequences are available and which substrates they have been shown to accept. A measure of database completeness for each enzyme type is captured through both a score for the percentage of papers with complete data entry and a user-entered score for how well the literature has been searched for that enzyme type (Figure 2 and Supplementary Figure 5). Importantly, the literature search score decreases over time, as this becomes more out of date.

Several initiatives are currently being developed to better record the data generated in biocatalysis and enzymology experiments upon publication, with the aim of both improving reproducibility and ensuring that data is recorded in a machine-readable format.^28−31^ As these approaches mature, and the deposition of data during publication becomes standard, the data generated by these approaches can be directly integrated into RetroBioCat-DB.

### Analysis of Biocatalysis Data

#### Exploring the Enzyme Toolbox

**Figure 3:**
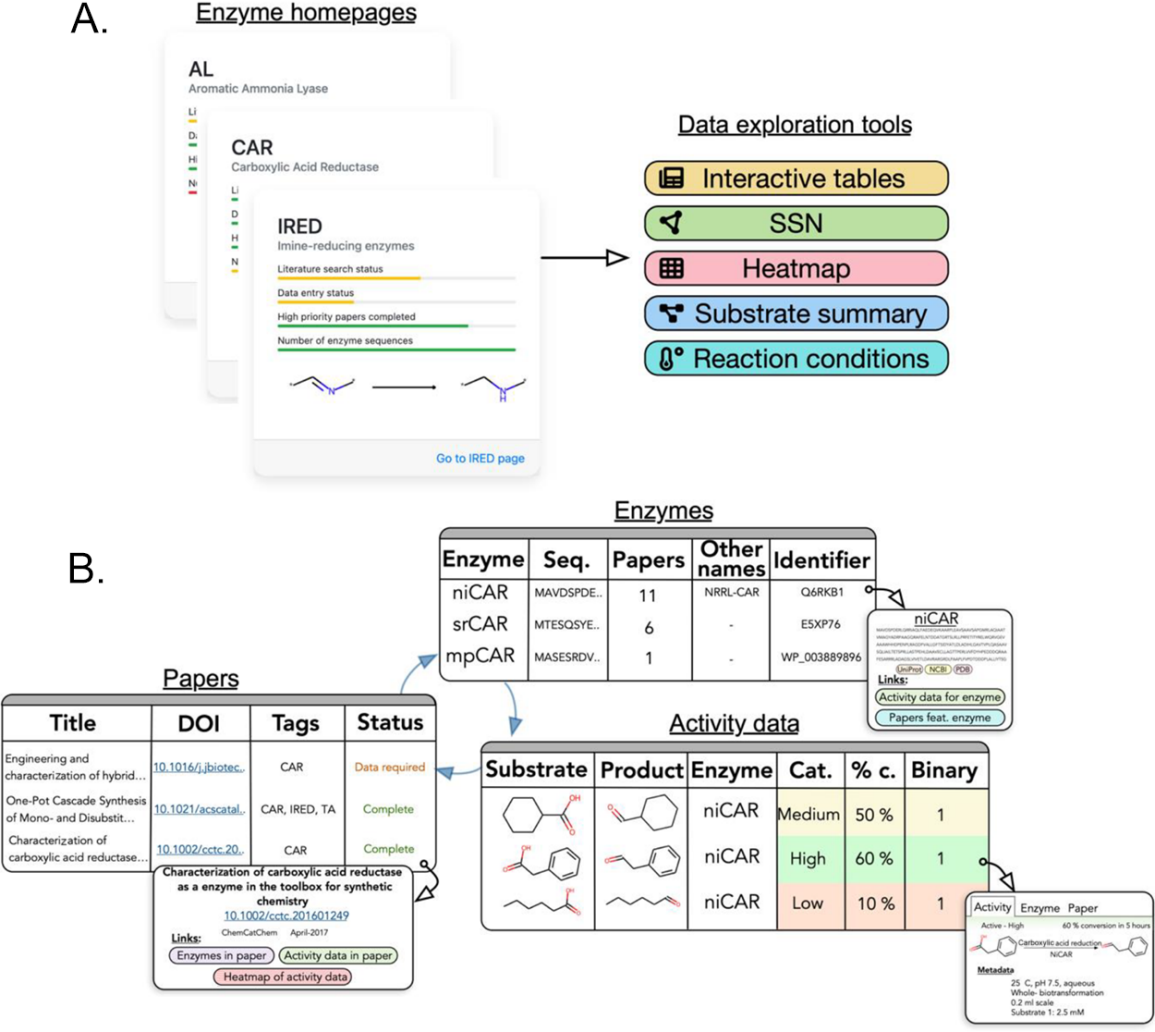
Exploring the enzyme toolbox. (A) Enzymes available in the database are listed on the main enzyme page and ordered according to the amount of data currently available for each enzyme class. Each enzyme has its own enzyme homepage, which acts as a launch pad for various interactive data exploration and visualization options. (B) Data in RetroBioCat-DB can be explored through a set of three tables, showing enzymes, activity data, or papers, respectively. A simplified representation of the tables is shown. For each table, clicking an individual row launches a pop-up window with more detailed information for that entry, which in turn can be used to launch queries targeting specific information of interest to the user. For example, the papers or activity data featuring a given enzyme can be accessed from the enzyme table.

Analysis of the biocatalysis data available in RetroBioCat-DB begins at the enzyme toolbox page, which lists each enzyme class in order of data availability. Status bars illustrate the completeness of the database in each case (Figure 3A). Selection of an enzyme class opens its enzyme homepage, which acts as a launch pad for the tables, visualization, and other analysis tools available (Figure 3A). In addition, further statistics about that enzyme class such as data entry completion, users in the curation team, reactions catalyzed, the number of unique products, and the enzyme sequences available are also available.

Three interlinked tables for enzyme sequences, activity data, and the papers from which this information is curated can be used to explore the data held in RetroBioCat-DB (Figure 3B). Tables are accessible from the enzyme homepage for each enzyme class. Importantly, each table can be rapidly filtered or sorted to access specific information (Supplementary Figure 17). Within each table, clicking on a row of interest launches a pop-up window with further information and links to related data. Related tables, such as the enzyme sequences reported in a specific paper, can be accessed through these pop-up windows, linking the tables together (Figure 3B).

A molecular similarity search tool is available to identify reactions with similar products to a query reaction (Supplementary Figure 18), as is available through the RetroBioCat synthesis planning tools. Indeed, this feature was recently put to good use in identifying ene-reductases (EREDs) during the development of a cascade.^32^ Other case studies for how the database enables enzyme identification during reaction design are available in the Supporting Information (Supplementary Figures 20–22). Furthermore, BLAST searches can be carried out against RetroBioCat-DB to identify characterized homologous sequences to a sequence of interest (Supplementary Figure 19), which can be particularly useful when making claims about the diversity of a novel biocatalyst against existing examples.^33^

#### Exploring Biological Context Using Sequence Similarity Networks (SSNs)

**Figure 4:**
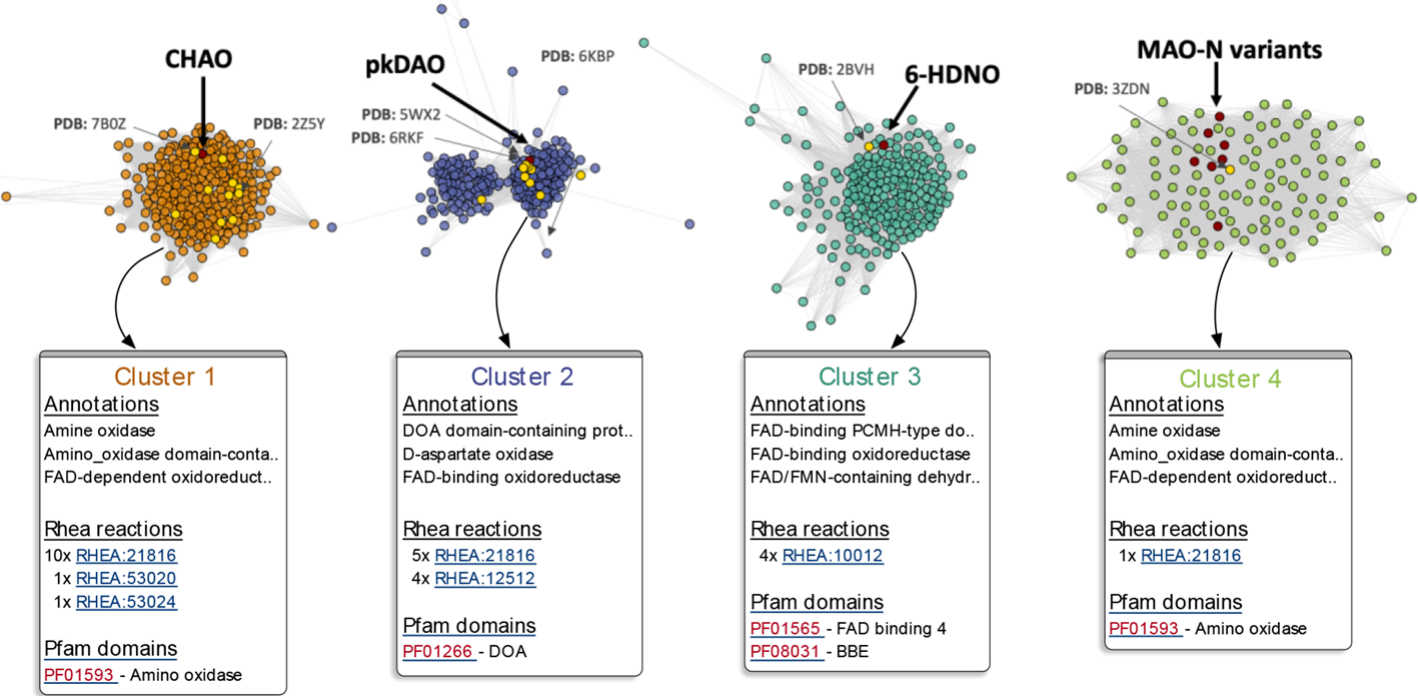
SSN for amine oxidase (AmOx) enzymes on RetroBioCat. Each node represents either a database sequence (shown in dark red and labeled in the figure) or a UniRef50 cluster. UniRef50 clusters with a representative sequence marked as annotated in SwissProt are shown in yellow. Edges between nodes are determined by their alignment score. The interactive SSN offers analysis of the clusters formed in the SSN, showing common annotations in UniProt, associated Rhea reactions, and Pfam domains.

To analyze the relationships between the enzymes captured in RetroBioCat-DB and to link to existing protein databases, interactive SSNs are automatically generated for each enzyme class (Figure 4). SSNs show how sequences from across the literature are related. Furthermore, displaying characterized enzymes alongside uncharacterized UniRef50 sequences allows areas of uncharacterized sequence space to be easily identified for future studies. The associated pfam domains, rhea reactions, pdb structures, and biological annotations from each UniRef50 sequence are captured and summarized in the SSN, either for each node or for each cluster presented in the SSN (Figure 4).

**Figure 5:**
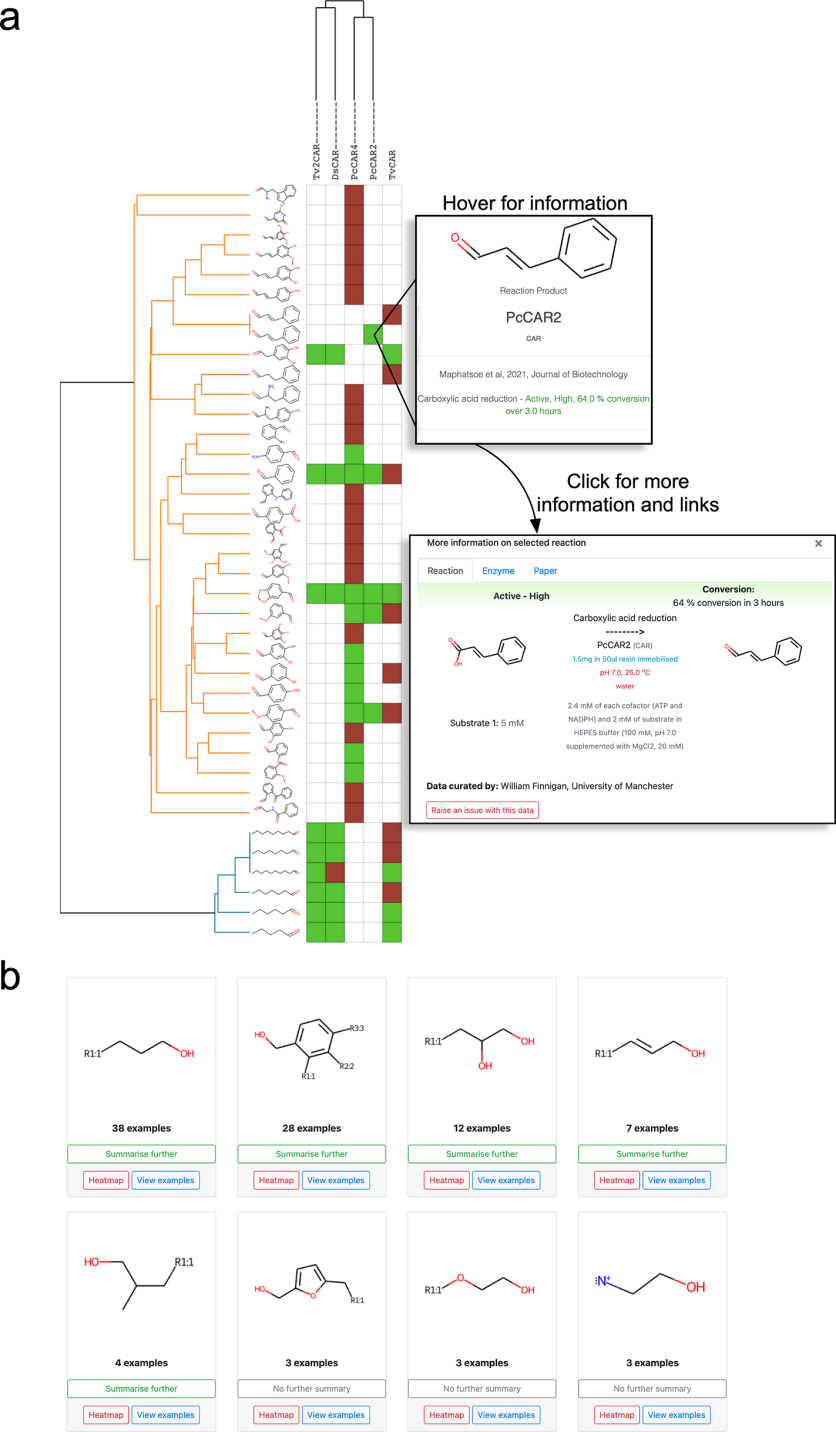
Visualizing substrate specificity. (A) Heatmap of activity data for fungal CARs showing reaction products, selected using the SSN tool. Green squares indicate one or more reports of activity, red indicates reports of no activity, and white spaces are pairings which are not in the database. Each square can be selected to launch a pop-up with more information on the reaction, with links to other information such as the enzyme sequence and the paper in which the activity was reported. Molecules and enzymes are grouped together and ordered using agglomerative clustering, generating dendrograms in both cases. (B) Substrate summary view for a selection of alcohol oxidase (AlOx) substrates. The number of molecules each core represents is shown as the number of examples. Each core can be summarized further or explored as a heatmap or a grid of examples.

SSNs have been utilized in numerous biocatalysis studies.^5,34,35^ Indeed, the use of SSNs has been popularized primarily through the availability of the genomic enzymology tools provided by the EFI,^17^ which we encourage the use of for more bespoke analysis. In contrast, RetroBioCat-DB SSNs are available for immediate analysis, putting the relevant biological context for a biocatalyst at a scientist’s fingertips. In addition, groups of related sequences can be selected for further analysis, such as through an activity heatmap (Figure 5).

#### Capturing Substrate Scope through Interactive Heatmaps and Substrate Summaries

Heatmaps are a powerful visualization tool for the analysis of data in two dimensions, such as activity data for enzyme and compound pairings. Indeed, heatmaps have been used for this purpose in several biocatalysis studies and reviews.^36,37^ RetroBioCat-DB offers a dynamic heatmap view, produced on demand for any dataset. For example, heatmaps can be created for all the activity data for a given enzyme class, created for the data in a single paper, or created for a specific selection of enzymes taken from an SSN, such as the fungal CARs shown in Figure 5A. Crucially, each heatmap is interactive, allowing the underlying data to be further interrogated.

Often substrate scope is presented using core structures for several examples with various R group substitutions.^7^ Presentation of data in this way can give scientists a simple way to understand the types of molecules which might be accepted by an enzyme. RetroBioCat-DB provides a substrate summary view which uses a Hasse diagram as reported by Heid et al. to automatically group substrates into a hierarchical structure for analysis.^18^ The substrate summary view shows core structures with R groups and the number of examples these represent, with an ability to select cores for further exploration (Figure 5B). Groups of molecules can be viewed in a grid, used to launch an activity table view, or sent to the heatmap for visualization.

#### Identification of Reaction Conditions Using Summary Graphs

**Figure 6:**
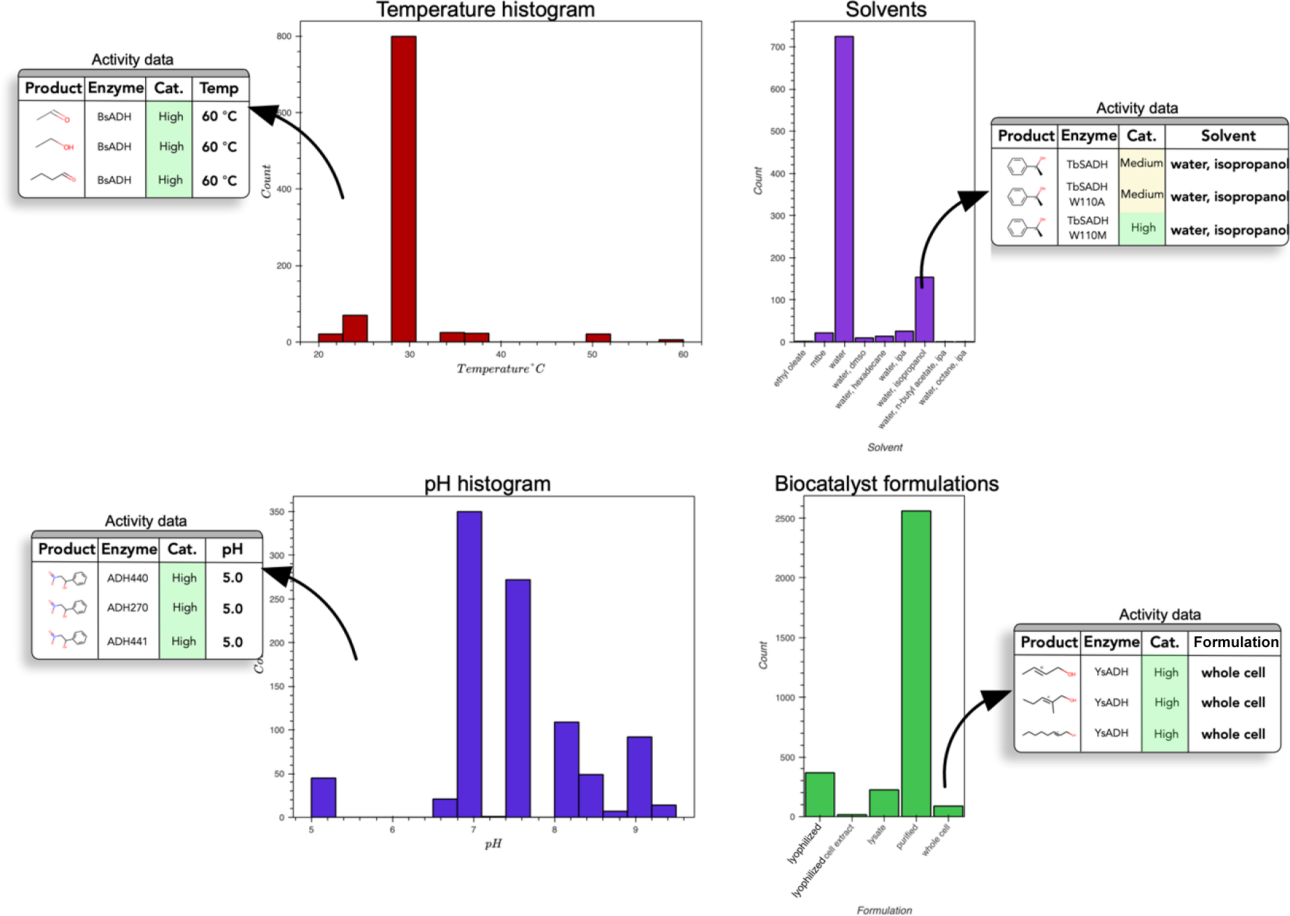
Reaction condition summary graphs for alcohol dehydrogenase (ADH) enzymes. Temperature and pH histograms show the distribution of conditions in the dataset, with the graphs linking to the activity data in each bin. Solvent and biocatalyst formulation bar graphs similarly show the frequency of each choice, again linking to the relevant activity data.

Selection of suitable reaction conditions is a critical step in designing biocatalytic reactions or cascades. RetroBioCat-DB provides a reaction condition overview page, which provides a summary of pH, temperature, solvent choice, and biocatalyst formulations recorded in the database for any given enzyme class and/or reaction (Figure 6). Each graph can be used to access the underlying information, for example, to select all the activity records at a certain pH or with a particular solvent.

Providing scientists with an overview of the conditions used in the literature offers a powerful tool to aid reaction design. The data captured in RetroBioCat-DB might also be used for automated reaction condition suggestion in the future. However, reliable predictions have been proven difficult to achieve in chemistry.^38^ Instead, data-driven human evaluation of the best reaction conditions offers a powerful middle ground.

## Conclusions

RetroBioCat-DB fills an important niche for biocatalysis data not met by other biology or chemistry databases (Supplementary Table 1). Furthermore, it provides a rich set of analysis tools allowing data to be interrogated in a myriad of ways and a publicly available data curation platform so that anyone can assist in adding new data to RetroBioCat-DB. A summary of the advantages and limitations of RetroBioCat-DB is available in Supplementary Table 2. A video giving an overview of how to use RetroBioCat-DB is also provided in the Supporting Information.

Identifying the feasibility of a proposed biocatalytic step is key to the success of biocatalysis synthesis planning.^4^ Making biocatalysis data easily accessible and interrogatable allows for a human to make informed decisions about which routes to pursue and which enzymes to utilize. RetroBioCat-DB enables enzyme selection in this way. However, reliable automated predictions on reaction feasibility and enzyme selection would further aid this process. Many exciting advances are being made using machine learning coupled with structural biology to predict activities for compound–protein combinations.^39,40^ The data captured in RetroBioCat-DB will be essential to effectively leverage these techniques in the future. For all data, the original paper can be accessed via its DOI link, allowing further exploration of additional information, methods, or analysis. While some articles may require a journal subscription, the rise in open access publishing will further democratize this knowledge in the future.

Beyond searching for an enzyme to carry out a specific reaction, RetroBioCat-DB offers a platform from which to explore broadly what an enzyme class is and what the enzymes within the class are capable of. Indeed, biocatalysis enzyme classifications can often span multiple pfam domains and reaction mechanisms, with naming driven by the chemistry catalyzed. The SSN tool can be used to shed light on the biological variety within an enzyme class, relating biocatalysts back to a range of existing biological databases. Furthermore, biocatalysis enzyme classes are often presented alongside broad reaction schemes, yet the existing substrate scope is rarely so generous. The interactive visualizations provided by RetroBioCat-DB are critical to communicate the sorts of reactions which are possible with a given enzyme class.

As the biocatalysis toolbox continues to expand through enzyme discovery or even de novo design of new enzymes,^41−43^ new enzyme classes will need to be added, facilitated by a public suggestion portal. However, in some areas, the scope of the database is constrained. For example, we do not capture enzymes in the developing area of biocatalytic oligonucleotide synthesis,^44^ carbohydrate active enzymes which are well served by the existing databases,^45^ or all of the available P450 chemistries which remain a challenge.

Data curation will be an ongoing challenge accelerated by the semi-automated tools provided by the RetroBioCat-DB curation platform. While the initial data has been primarily provided by our group, the curation platform is openly available, and all contributions are attributed. We hope to further automate the curation process; however, human oversight will likely remain critical. Importantly, RetroBioCat-DB is an excellent resource for review articles, and data curators who have contributed to the database can request access to datasets for this purpose. Indeed, the database has already been put to good use in reviewing amide bond forming enzymes.^46^

As the inclusion of machine-readable datasets becomes more common in academic publishing,^29,30^ this should also greatly accelerate or even replace the curation process. An agreed-upon standard for reporting biocatalysis data, as has been achieved by STRENDA for enzymology and adopted as a recommendation by journals,^28^ could substantially increase the availability of machine-readable data. Furthermore, the re-use and easy discovery of data in this format (for example, via RetroBioCat-DB) should enhance the impact of published work, acting as an incentive for authors to publish standardized machine-readable datasets.

In summary, RetroBioCat-DB provides an important resource to the biocatalysis and chemistry communities, democratizing biocatalysis knowledge.

## Data Availability

The RetroBioCat database can be freely accessed at https://retrobiocat.com. Data from the database is available on request. The code is available at https://github.com/willfinnigan/retrobiocat-db.

## Supporting Information Available

Database structure; automated pipeline for SSN creation; workflow for adding papers by DOI; workflow for adding papers using keyword searches directly; paper search scores; paper ready for curation; overview tab; sequences tab; add enzyme window; edit sequence information; molecule addition by chemical name lookup; molecule addition by manual drawing; molecule addition by excel upload; activity tab; excel upload of activity data; merging identical sequences; examples of the table views available; substrate specificity search; BLAST search; identification of HMO as a possible enzyme for the secondary alcohol oxidation step; identification of CAR enzymes for synthesis of 3-ethylbenzaldehyde; identification of S-selective reactions for an IRED reaction; comparison of scope, search, data exploration, and curation of biology and chemistry databases; and summary of the advantages and limitations of the RetroBioCat database (PDF)
RetroBioCat database video (MOV)

## Supplementary Material

cs3c01418_si_001.pdf

cs3c01418_si_002.mov
