# Supplementary material for: RetroBioCat Database: A Platform for Collaborative Curation and Automated Meta-Analysis of Biocatalysis Data
Source: ACS Catal. 2023 Aug 22;13(17):11771–80. doi: 10.1021/acscatal.3c01418 (PMC10476152; doi:10.1021/acscatal.3c01418)
Supplement: Supplementary file 1 — cs3c01418_si_001.pdf [file cs3c01418_si_001.pdf]

The RetroBioCat Database - a platform for collaborative curation and automated meta-analysis of biocatalysis data

William Finnigan[1], Max Lubberink[1], Lorna J. Hepworth[1], Joan Citoler[1], Ashley P Matthey[1], Grayson J. Ford[1], Jack Sangster[1], Sebastian C. Cosgrove[1], Bruna Zucoloto da Costa[1], Rachel S. Heath[1], Thomas W Thorpe[1], Yuqi Yu[1], Sabine L. Flitsch[1]* & Nicholas J. Turner[1]*

[1] Department of Chemistry, Manchester Institute of Biotechnology, University of Manchester, 131 Princess Street, M1 7DN, Manchester, UK

* To whom correspondence should be addressed. Email: nicholas.turner@manchester.ac.uk
* Correspondence may also be addressed to. Email: sabine.flitsch@manchester.ac.uk,

Table of Contents

1 Database structure 3

2 Paper searching and addition 4

- 2.1 Paper addition by DOI 4
- 2.2 Paper addition by key word search 5
- 2.3 Literature search score 6

3 Curation platform 6

- 3.1 Self-assignment of papers for curation 6
- 3.2 Curation portal - Overview tab 7
- 3.3 Curation portal - Sequence tab 7
- 3.4 Curation portal - Molecules tab 9
- 3.5 Curation portal - Activity tab 10
- 3.6 Handling duplicate sequence entries 12

4 Analysis platform 13

- 4.1 Tables 13
- 4.2 Searches 14

5 Database comparison tables 16

6 Enzyme identification case studies 19

- 6.1 Clodropigrel – AlOx enzyme identified during synthesis planning 19
- 6.2 Identification of a CAR enzyme for the synthesis of 3-ethylbenzaldehyde using the similarity search tool 20
- 6.3 Identification of an S selective IRED enzyme for the synthesis of (2S)-2-phenylpiperidine using the similarity search tool 21

7 References 21

1 Database structure

The database consists of five core interlinked tables (**Supplementary Figure 1**). This data model allows us to capture, relate, and make queries on the critical information for biocatalysis enzymes. For example, we can access a list of the activity data reported in a specific paper, or for a specific enzyme, or for an enzyme type in general. A full breakdown of the fields in the database and how they relate is available in the supporting information. A particular challenge in curating biocatalysis activity data is the variety of ways in which this information can be collected and recorded. In general, one of five data types is possible: enzyme kinetics, specific activity, relative activity, percentage conversion, or a qualitative measure of activity from a colorimetric assay or similar. Fields to capture these data types are present in the activity table (**Supplementary Figure 1A**), along with fields for experimental meta-data. Relative activity is not included, as these measurements cannot be compared across publications. Where relative activity is reported, this can be converted either to a specific activity, or to a qualitative value, depending on whether the original benchmark measurement is available.

To facilitate comparison of different datatypes recorded in the literature, activity measurements can be converted in a cascade down from full enzyme kinetics to a binary measurement of active or inactive (**Supplementary Figure 1B**). For example, a theoretical specific activity can be calculated at a standard concentration of 10 mM from enzyme kinetics data, for comparison with other data. Likewise, conversions can be converted to categorical values of high, medium, or low according to predefined criteria (Supporting Information). These conversions are made automatically as data is entered.

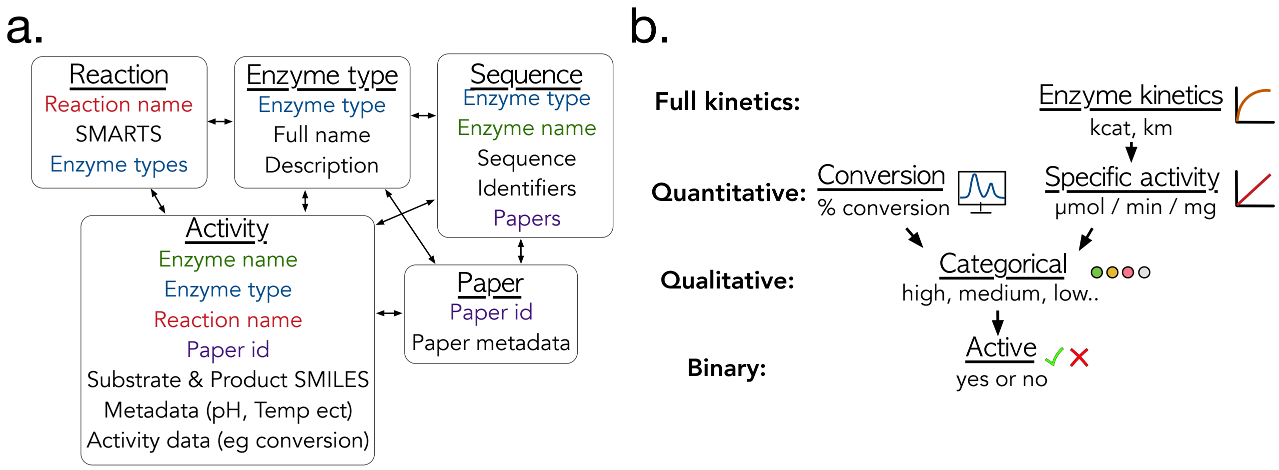

Supplementary Figure 1 – Database structure. A. The core data tables implemented in MongoDB, which hold the information in the database, and link to the reaction rules for RetroBioCat [1]. Arrows illustrate the links between the tables (see supporting information for further detail). **B.** Various types of activity data that can be reported in the biocatalysis literature. Data types can be converted in a cascade from enzyme kinetics through to a binary output for activity.

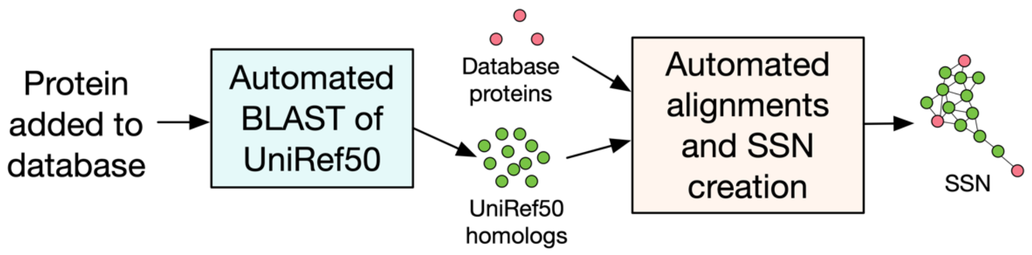

Supplementary Figure 2 – Automated pipeline for sequence similarity network (SSN) creation. As proteins are added to the database through the curation portal, and marked as reviewed, they are used to automatically BLAST the UniRef50 database to get homologs. Homologs and database sequences are subjected to all-by-all alignments to generate a SSN, which is the available to users.

2 Paper searching and addition

2.1 Paper addition by DOI

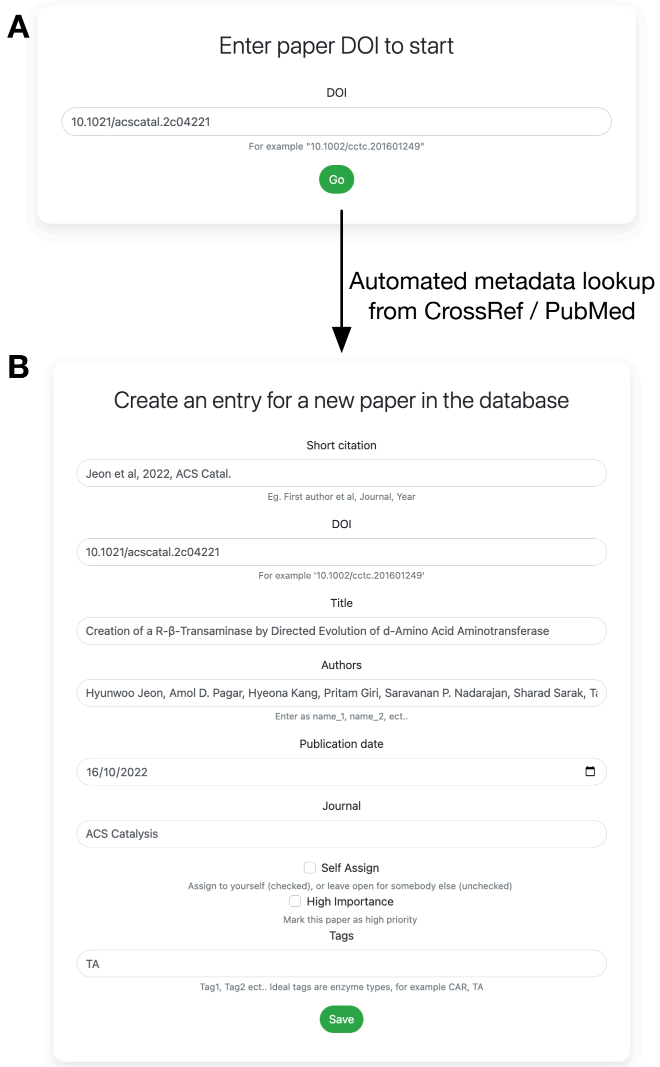

Supplementary Figure 3 – Workflow for adding papers by DOI. A. A DOI is entered and queried against the existing database to check if it is already present. **B.** If paper has not already been added, CrossRef and PubMed are searched to

retrieve the relevant metadata, with the option to manually alter these fields as necessary before saving the paper to the database.

2.2 Paper addition by key word search

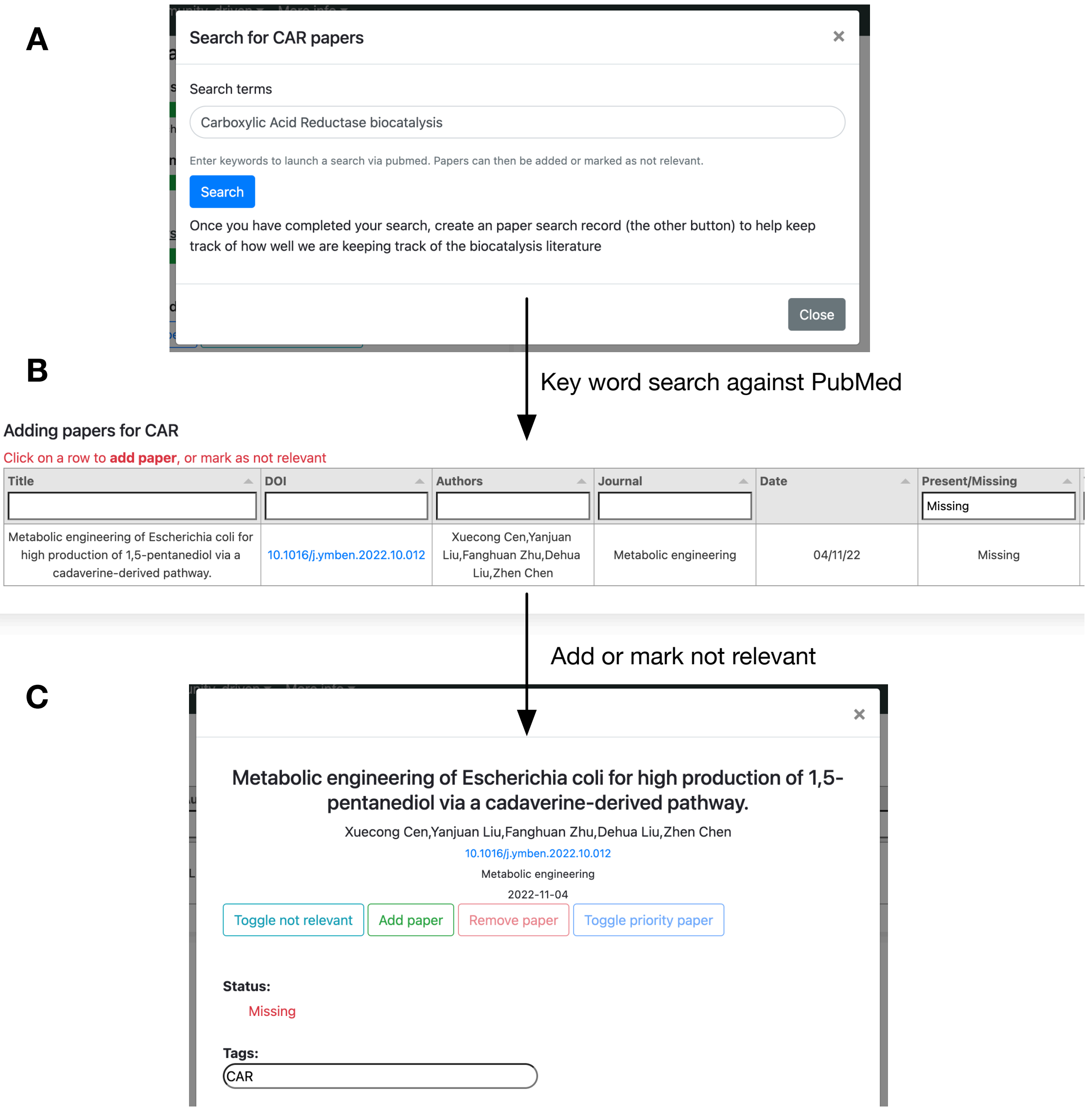

Supplementary Figure 4 – Workflow for adding papers using keyword searches directly. ***A.*** *Search terms are entered and used to programatically search PubMed.* ***B.*** *Results are returned as a table. Papers which are not already in the database are marked as missing, or not relevant.* ***C.*** *Clicking on a table row launches a dialog for that paper, allowing it to be added, or marked not relevant.*

2.3 Literature search score

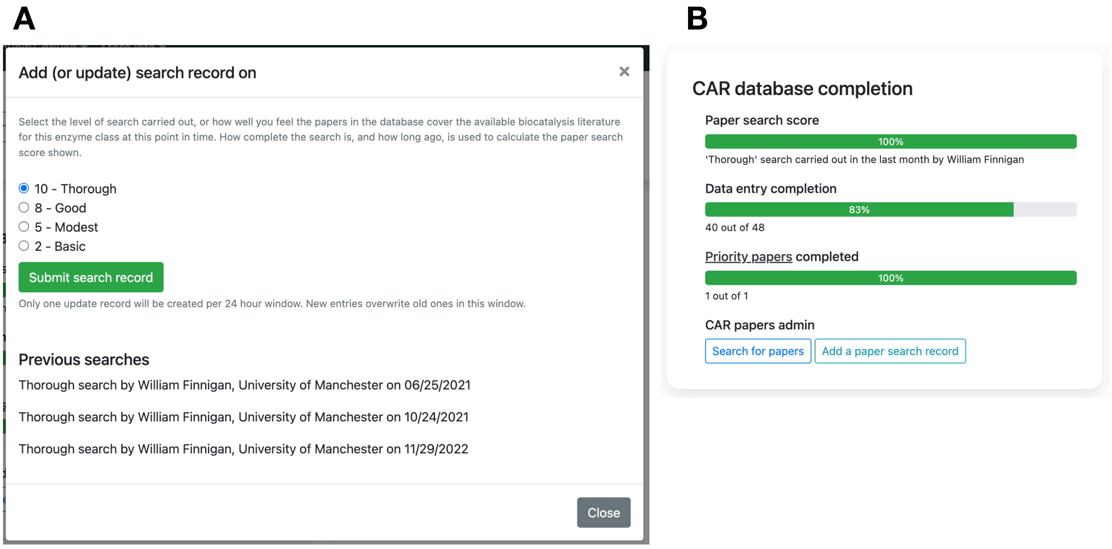

Supplementary Figure 5 – Paper search scores. A. Upon searching the literature for a given enzyme class, a record of how complete the search was can be made to update the search score for that enzyme. The score will decrease over time, to reflect the fact that new literature is published. **B.** The search score is displayed alongside the data entry completion and the number of priority papers completed.

3 Curation platform

3.1 Self-assignment of papers for curation

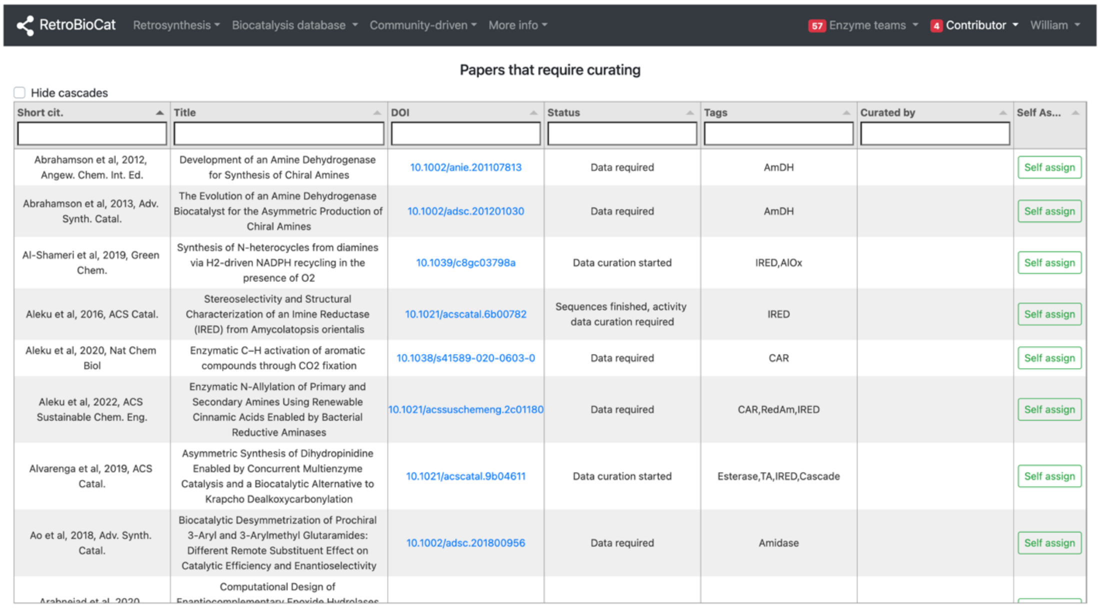

Supplementary Figure 6 – Paper ready for curation. Table showing some of the paper in the database not yet curated. Clicking 'Self assign' assigns that paper to the user and launches the curation portal.

3.2 Curation portal - Overview tab

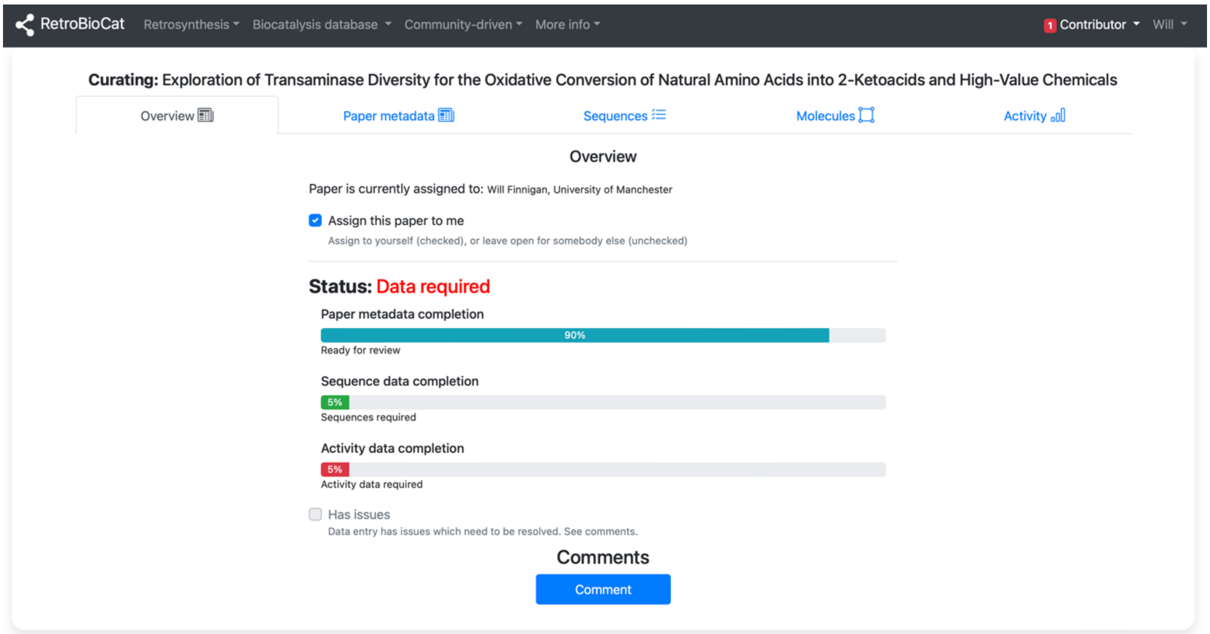

Supplementary Figure 7 – Overview tab. The first tab in the curation portal can be used to unassign the paper, write comments on the curation process, and shows the progress of the curation effort for that paper.

3.3 Curation portal - Sequence tab

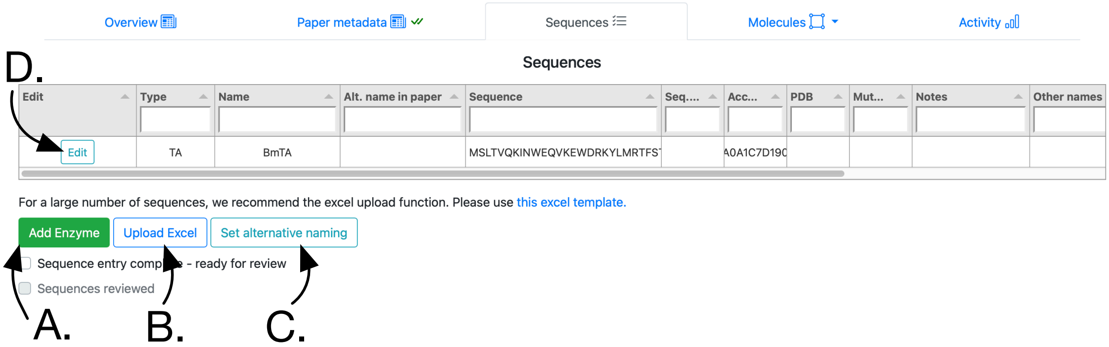

Supplementary Figure 8 – Sequences tab. The sequence tab allows the enzymes used in the paper to be added to the database. **A.** Enzymes are added by selecting the 'Add Enzyme' button, which launches the add enzyme window (**Supplementary Figure 9**). **B.** To add sequences in bulk, an upload excel method is also available. **C.** Where sequences have multiple names assigned to them, the use of an alternative as the default name can be selected for use when adding activity data. **D.** Each sequence database entry can be edited by clicking the corresponding "Edit' button, launching an edit window (**Supplementary Figure 10**).

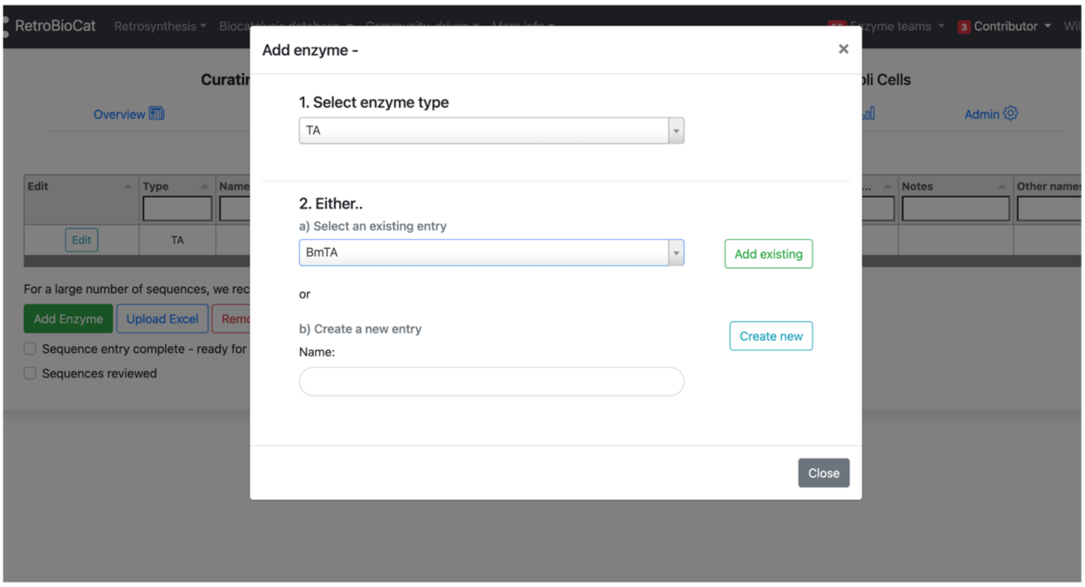

Supplementary Figure 9 – The add enzyme window. The enzyme type to be added must first be selected from the dropdown menu. Then either an enzyme previously added to the database of that type can be selected and added to this paper. Or, a new sequence entry can be created, provided an enzyme does not already exist with that name. Other information can later be added in the edit sequence window (**Supplementary Figure 10**).

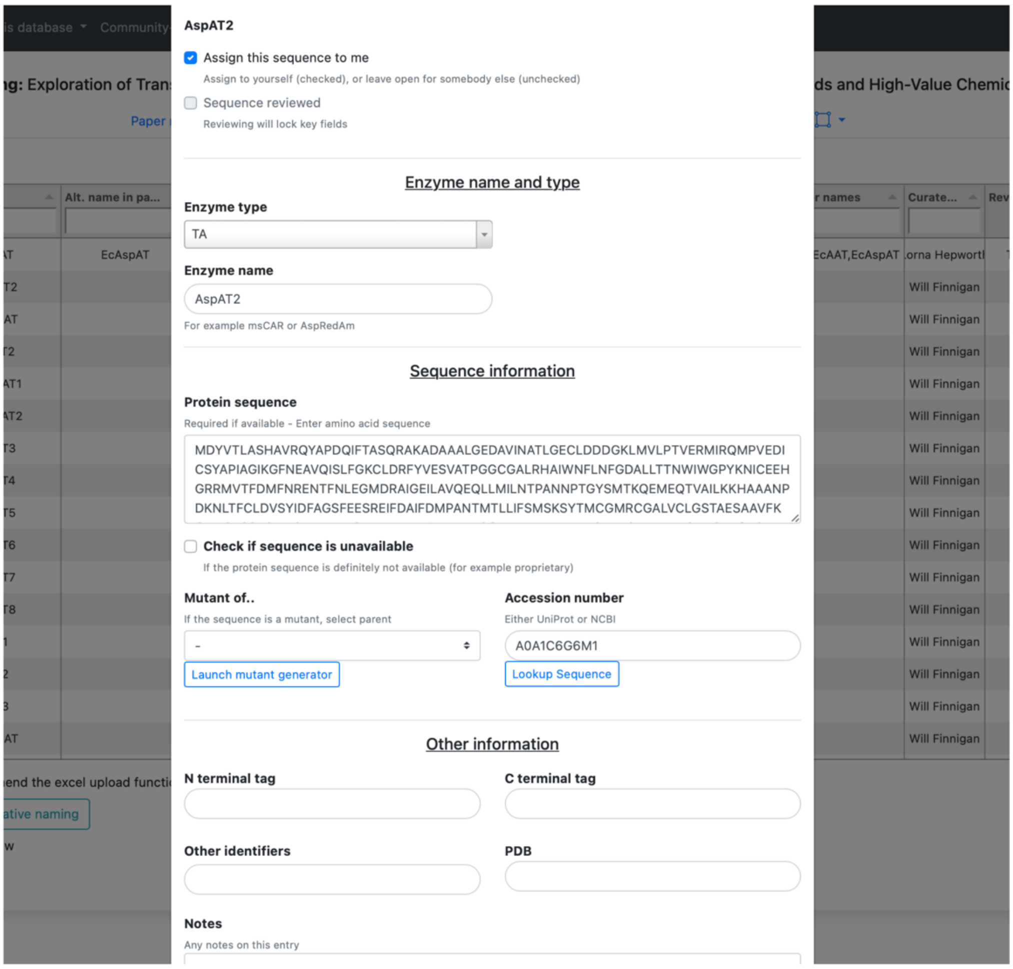

Supplementary Figure 10 – Edit sequence information. The 'Edit' button for each sequences launches an edit sequence information window. The protein sequence can be pasted directly, and is automatically validated when saving. DNA

sequences will automatically be translated to protein. Protein sequences which feature a recognised N or C terminal tag will automatically have this tag transferred to the relevant field. Alternatively, an accession number can be used to lookup the sequence from both UniProt and the NCBI database. Other fields can be entered as necessary.

3.4 Curation portal - Molecules tab

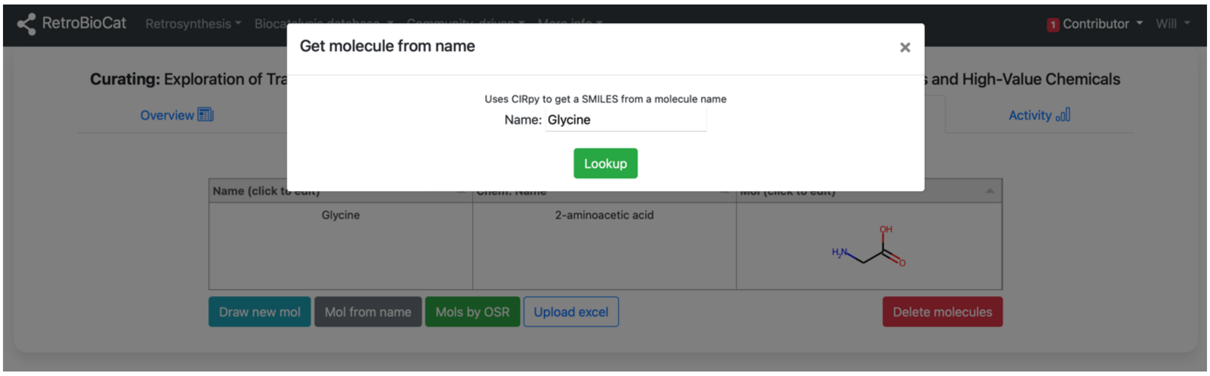

Supplementary Figure 11 – Molecule addition by chemical name lookup. Molecules can be added by using chemical names to retrieve SMILES strings. In the example Glycine is entered, retrieving the correct molecule.

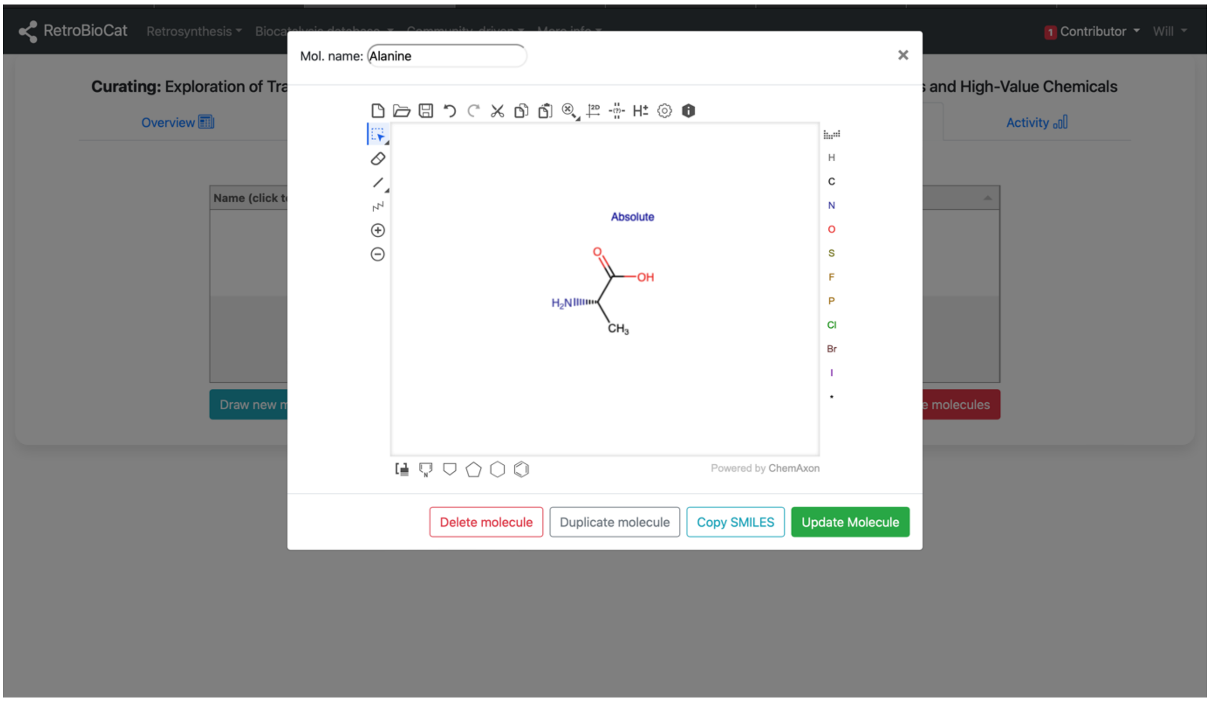

Supplementary Figure 12 – Molecule addition by manual drawing. Molecules can simply be drawn manually to add them. The same interface can be used to edit molcules, or make corrections to molecules added by other automated means.

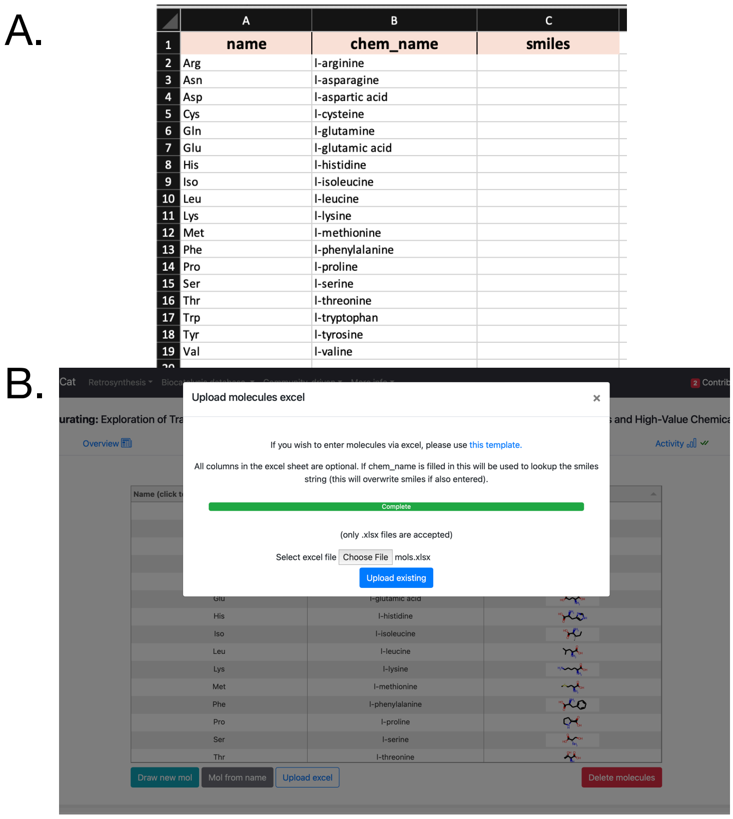

Supplementary Figure 13 – Molecule addition by excel upload. Molecules can be added in bulk by uploading an excel sheet. In the example shown, chemical names are used to look-up the relevant SMILES in each case. **A.** Example of an excel sheet for upload. **B.** Result after successful upload.

3.5 Curation portal - Activity tab

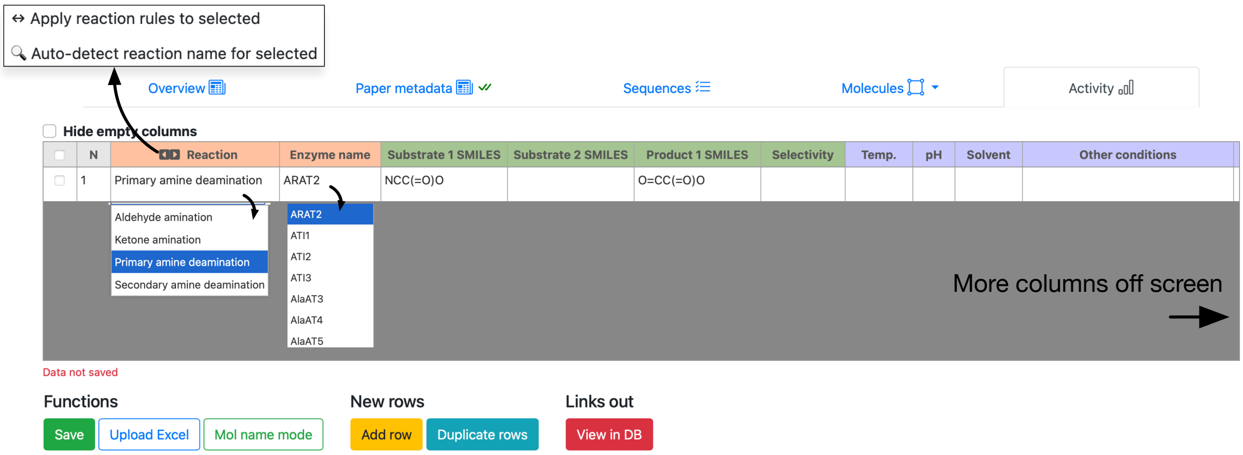

Supplementary Figure 14 – The activity tab. Activity data can be entered directly into the table provided in the activity tab, before saving this to the database using the save button. A series of automated checks must be passed to allow saving, such as valid SMILES being entered. Reactions and enzyme names will auto complete from the available sequences, entered in the sequences tab. Indeed, only enzymes in the sequences tab are valid in this column. The reaction column also features a button to allow the RetroBioCat reaction rules combined with either the substrates or products in the table to either automatically detect which reaction should be entered, or to apply that reaction to generate the corresponding substrates or product. Molecules can be entered by naming by switching to Mol name mode, which allows molecules entered in the molecules tab to be selected, loading the relevant SMILES in each case.

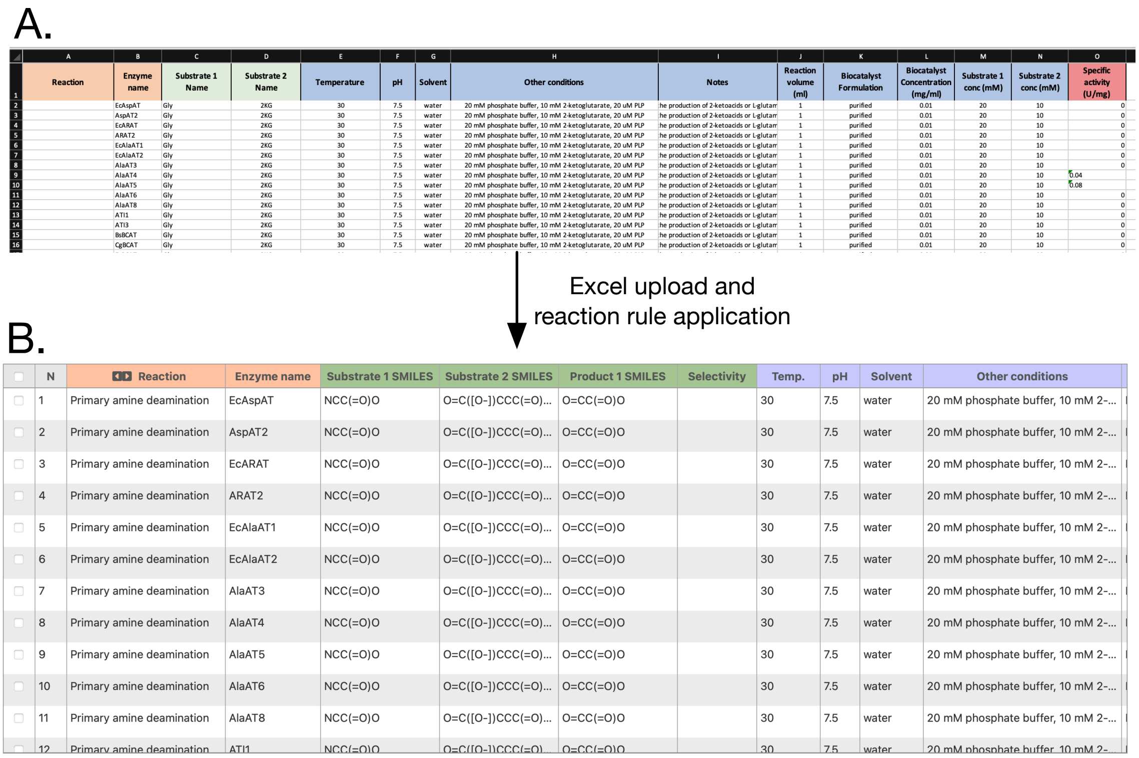

Supplementary Figure 15 – Excel upload of activity data. For large datasets, it can be helpful to compile the relevant information offline and then upload it. Shown is an example of this process. In particular, molecules entered in the molecules tab can be used in the excel, which upon uploading will automatically generate the required SMILES strings, as shown in the example.

3.6 Handling duplicate sequence entries

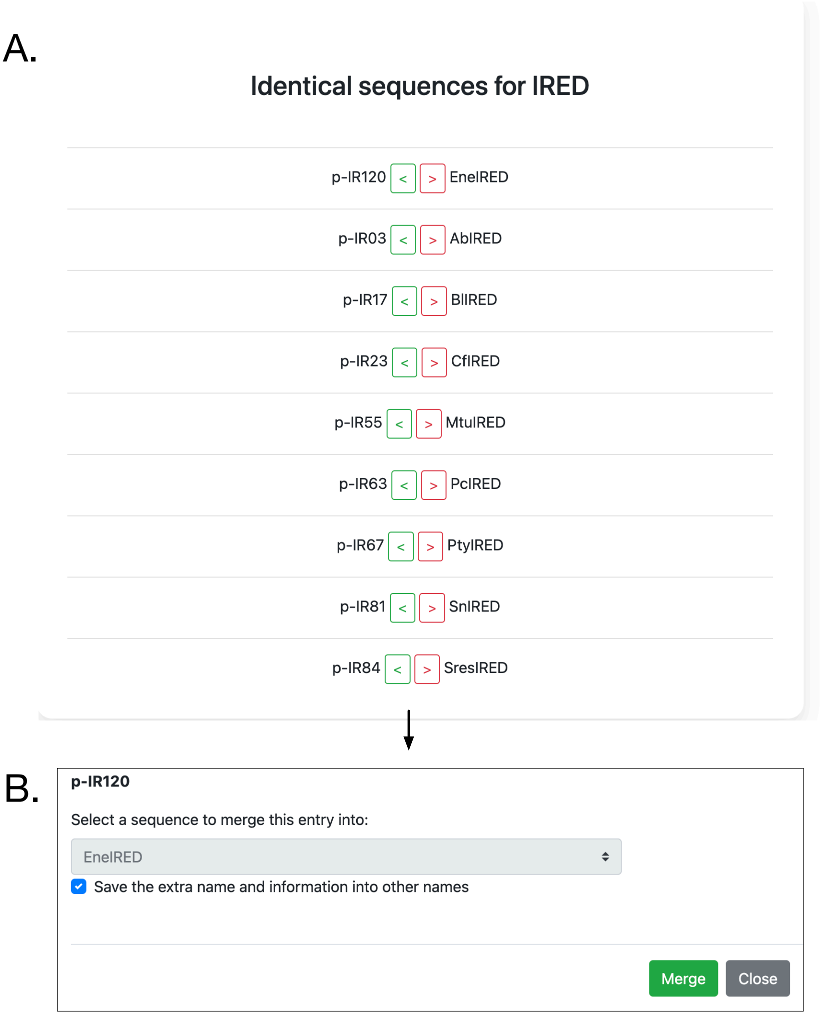

Supplementary Figure 16 – Merging identical sequences. A. Sequences which have identical amino acid sequences are identified as matching, with options to merge one entry into another. This platform is only available to admins. **B.** An example of the window for merging two enzyme sequences is shown. The name of the sequence being merged will be saved in the 'Other names' field of the sequence being kept.

4 Analysis platform

4.1 Tables

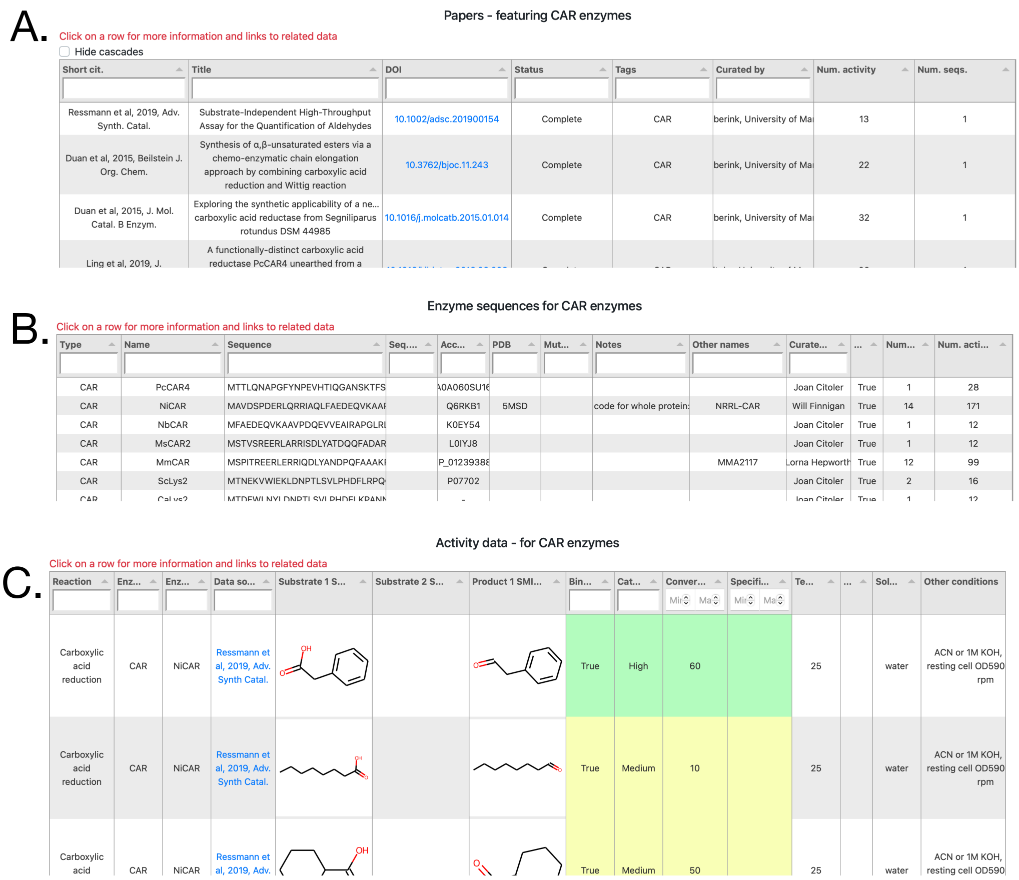

A. Papers - featuring CAR enzymes

Click on a row for more information and links to related data

Hide cascades

Short cit.	Title	DOI	Status	Tags	Curated by	Num. activity	Num. seqs.
Ressmann et al, 2019, Adv. Synth. Catal.	Substrate-Independent High-Throughput Assay for the Quantification of Aldehydes	10.1002/adsc.201900154	Complete	CAR	berink, University of Ma	13	1
Duan et al, 2015, Beilstein J. Org. Chem.	Synthesis of α,β-unsaturated esters via a chemo-enzymatic chain elongation approach by combining carboxylic acid reduction and Wittig reaction	10.3762/bjoc.11.243	Complete	CAR	berink, University of Ma	22	1
Duan et al, 2015, J. Mol. Catal. B Enzym.	Exploring the synthetic applicability of a ne... carboxylic acid reductase from Segniliparus rotundus DSM 44985	10.1016/j.molcatb.2015.01.014	Complete	CAR	berink, University of Ma	32	1
Ling et al, 2019, J. [illegible]	A functionally-distinct carboxylic acid reductase PcCAR4 unearthed from a [illegible]	[illegible]	[illegible]	[illegible]	[illegible]	[illegible]	[illegible]

B. Enzyme sequences for CAR enzymes

Click on a row for more information and links to related data

Type	Name	Sequence	Seq....	Acc...	PDB	Mut...	Notes	Other names	Curate...	...	Num...	Num. acti...
CAR	PcCAR4	MTTLQNAPGFYNPEVHTIQGANSKTFS		A0A060SU16					Joan Citoler	True	1	28
CAR	NiCAR	MAVDSPDERLQRRIAQLFAEDEQVKAAI		Q6RKB1	5MSD		code for whole protein:	NRRL-CAR	Will Finnigan	True	14	171
CAR	NbCAR	MFAEDEQVKAAVPDQEVVEAIRAPGLRI		K0EY54					Joan Citoler	True	1	12
CAR	MsCAR2	MSTVSREERLARRISDLYATDQQFADAR		L0IYJ8					Joan Citoler	True	1	12
CAR	MmCAR	MSPITREERLERRIQDLYANDPQFAAAKI		P_01239388				MMA2117	Lorna Hepworth	True	12	99
CAR	ScLys2	MTNEKVWIEKLDNPTLSVLPHDFLRPQ		P07702					Joan Citoler	True	2	16
CAR	CaLys2	MTDEWLNYLDNPTLSVLPHDFLKPANN		-					Joan Citoler	True	1	12

C. Activity data - for CAR enzymes

Click on a row for more information and links to related data

Reaction	Enz...	Enz...	Data so...	Substrate 1 S...	Substrate 2 S...	Product 1 SMI...	Bin...	Cat...	Conver...	Specifi...	Te...	...	Sol...	Other conditions
Carboxylic acid reduction	CAR	NiCAR	Ressmann et al, 2019, Adv. Synth Catal.				True	High	60		25		water	ACN or 1M KOH, resting cell OD590 rpm
Carboxylic acid reduction	CAR	NiCAR	Ressmann et al, 2019, Adv. Synth Catal.				True	Medium	10		25		water	ACN or 1M KOH, resting cell OD590 rpm
Carboxylic acid [illegible]	CAR	NiCAR	Ressmann et al, 2019, Adv. [illegible]				True	Medium	50		25		water	ACN or 1M KOH, resting cell OD590 [illegible]

Supplementary Figure 17 – Examples of the table views available. A. Papers table. The example shows papers which feature a CAR. **B.** Enzymes table. The example shows CAR enzymes. **C.** Activity table. The example shows all activity data for CAR enzymes. For all three tables, cicking on a row launches a pop-up with further information and links to additional data.

4.2 Searches

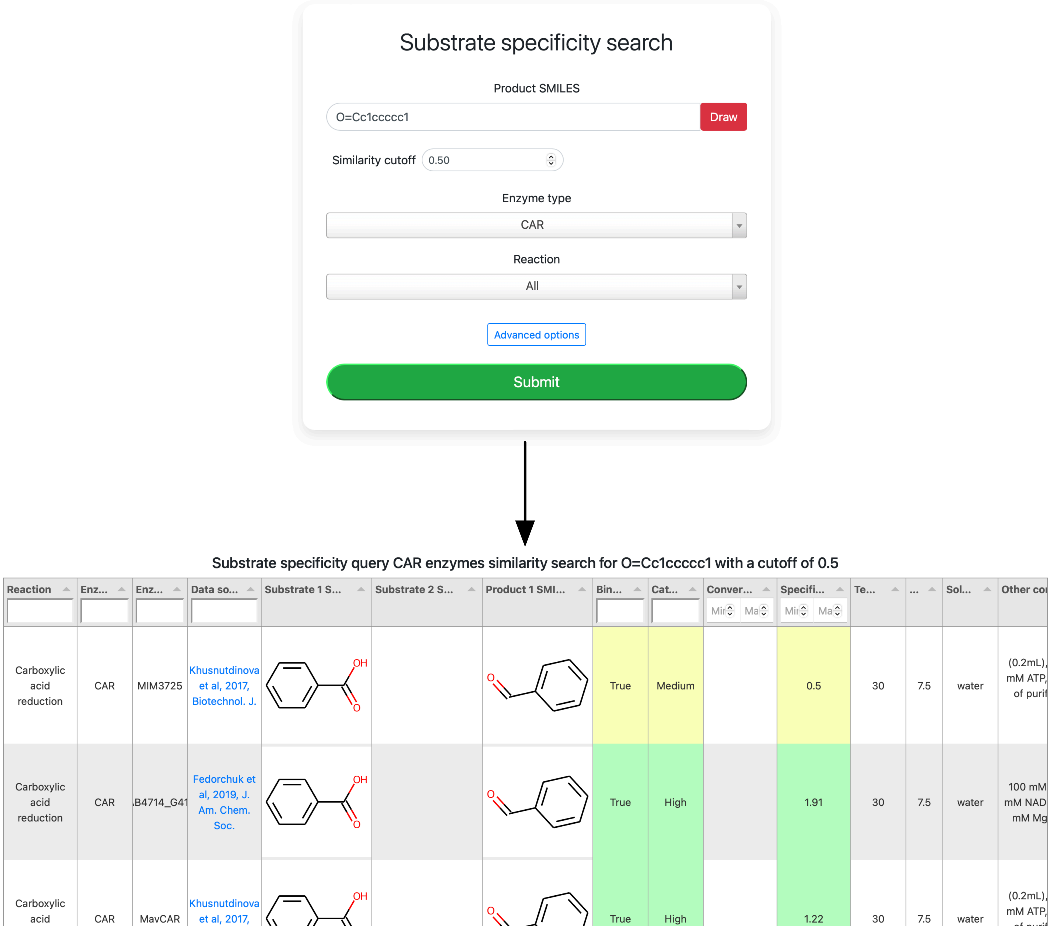

Supplementary Figure 18 – Substrate specificity search. The substrate specificity search page allows a user to enter a SMILES or draw a molecule, which is used to search for similar molecules in the database. Searches can be restricted by enzyme type and/or reaction. The standard RDKit fingerprints are used, with the tanimoto similarity coefficient. The result of the similarity search launches an activity table similar to as shown in Supplementary Figure 17.

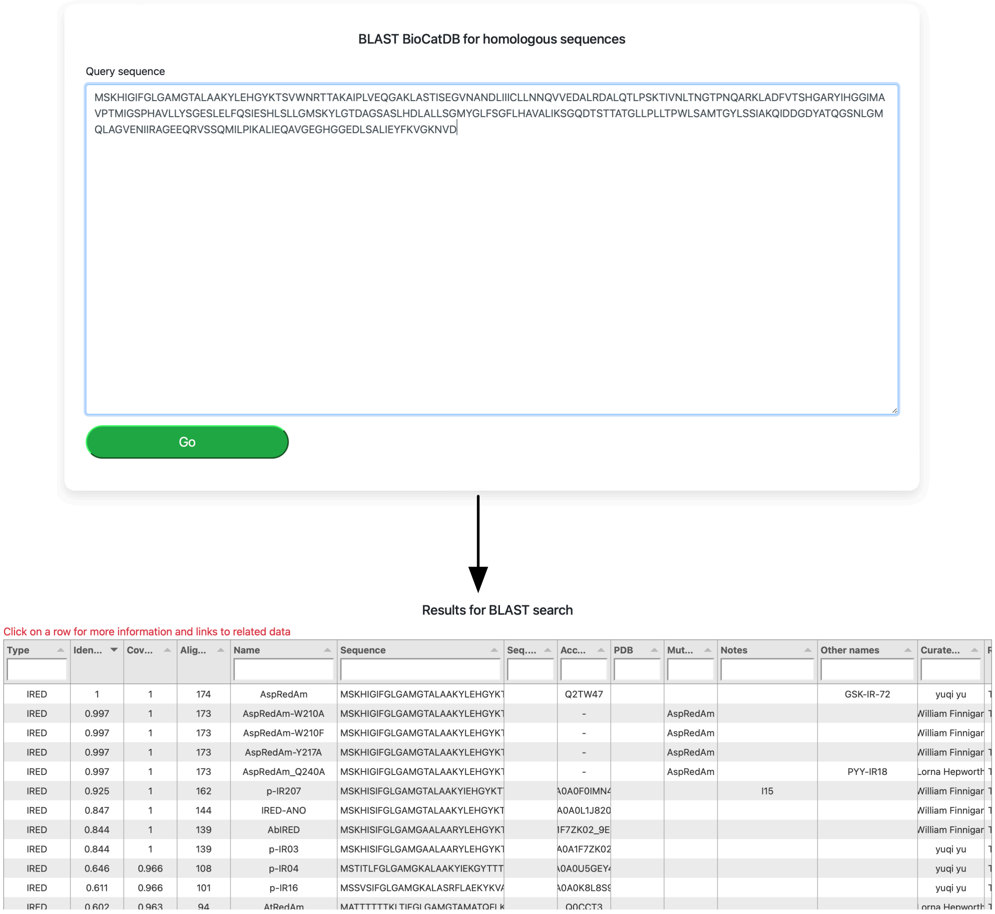

Supplementary Figure 19 – BLAST search. All of the enzyme sequences in the RetroBioCat database can be searched using a BLAST search. A protein sequence is entered, aligned with all the sequences, and the results returned in an enzyme table similar to as shown in in Supplementary Figure 17. Data can be sorted as required using the table headings.

5 Database comparison tables

Supplementary Table 1 – A comparison of scope, search, data exploration and curation of biology and chemistry databases				
**Database**	**Scope**	**Search and data exploration**	**Data curation**	**Access**
RetroBioCat-DB (this work)	Focus on enzymes in the biocatalysis toolbox, as they are described in the biocatalysis literature.	Structure-based searches, sequence-based searches, enzyme name lookup, interactive data analysis tools and tables.	Openly accessible, streamlined curation platform, with review by the maintainers of RetroBioCat.	Open
BRENDA [2]	Enzyme functional data as categorised by EC numbers.	Keyword and database identifier searches. Structure based search is also available.	Private curation by maintainers of BRENDA. Users can submit curation requests.	Open
Rhea [3]	Biochemical reactions primarily focused on metabolism	Keyword and database identifier searches.	Private curation by the Swiss Institute of Bioinformatics.	Open
KEGG [4]	Biochemical reactions primarily focused on metabolism	Keyword and database identifier searches.	Private curation by maintainers of KEGG.	Open
UniProt [5]	Large database of protein sequences and related information.	Keyword and database identifier searches. BLAST search also available.	Private manual curation by EMBL.	Open
Biocatnet [6]	Primarily protein sequences for a select group of biocatalysis enzymes, organised by family.	BLAST search. Tables of protein sequences grouped into families.	Sequence datasets added on a publication-by-publication basis by the maintainers.	Open
SabioRK [7]	Biochemical reactions and kinetic rate equations. Organised by EC number.	Keyword and database identifier searches.	Private curation by maintainers of SabioRK. Users can submit curation requests.	Open
STRENDA-DB [8]	Enzymology database for STRENDA, holding data on enzyme kinetics.	Keyword and database identifier searches.	Users submit their own data.	Open

PrenDB [9]	Prenytransferase enzymes and reactions.	Browse tables of substrates, products, cofactors, or enzymes. Predictions for prenylation.	Private curation by the authors of PrenDB.	Open
CAZy [10]	Carbohydrate-Active enZYmes. Describes the families of structurally-related catalytic and carbohydrate-binding modules. Primarily sequence information.	Browse by family classification.	Private curation by CAZy maintainers.	Open
Redoxibase [11]	Database of oxido-reductase superfamilies encoding sequences	Keyword searches	Private curation by Redoxibase contributors.	Open
Reaxys	Commercial database and synthesis planning tools for organic chemistry.	Keyword search, structure-based search, query builder.	Private curation by Reaxys	Commercial
Scifinder	Commercial database and synthesis planning tools for organic chemistry.	Keyword search, structure-based search,	Private curation by Scifinder	Commercial

Supplementary Table 2 – Summary of the advantages and limitations of the RetroBioCat database.
**Advantages**<br>- Naming of enzyme classes reflecting use in the biocatalysis literature.<br>- Data on activity against synthetic substrates not captured in other databases.<br>- Structure-based searching of molecules using similarity.<br>- Sequences linked to activity data (where sequences are available)<br>- Intuitive and easy-to-use interface allowing quick navigation between sequence, activity, and literature source.<br>- Publicly available curation platform with review system<br>- Tools to globally explore substrate and sequence space for an enzyme class.
**Limitations**<br>- Focus on a smaller selection of biocatalysis enzymes, as opposed to all enzymes.<br>- Curation is ongoing, some enzyme classes are not yet well covered.<br>- Curation of data is still time-consuming. There is scope for further automation.<br>- Free text fields for some of the activity metadata are less amenable to analysis (but necessary to provide flexibility to cover all cases).<br>- Some areas of biocatalysis are not covered, such as biocatalytic oligonucleotide synthesis, carbohydrate active enzymes, or all the available P450 chemistries.

6 Enzyme identification case studies

6.1 Clodropigrel – AlOx enzyme identified during synthesis planning

Using the synthesis planning capabilities of RetroBioCat [1], a biocatalytic cascade to Clodropigrel can be planned consisting of an alcohol oxidase (AlOx) and a reductive aminase (RedAm). Any similar reactions in the RetroBioCat database are automatically identified during synthesis planning, resulting in a green reaction node (**Supplementary Figure 20 – A**). The user can then click on this to get more information, including information about the similar reaction such as conversion and reaction conditions, data on the enzyme including its protein sequence (if available) and links to other databases such as UniProt, and the paper this reaction was reported in (**Supplementary Figure 20 – B**). The AlOx reaction shown is quite similar, the only differences being the additional chlorine atom and the presence of an ester group in place of a carboxylic acid.

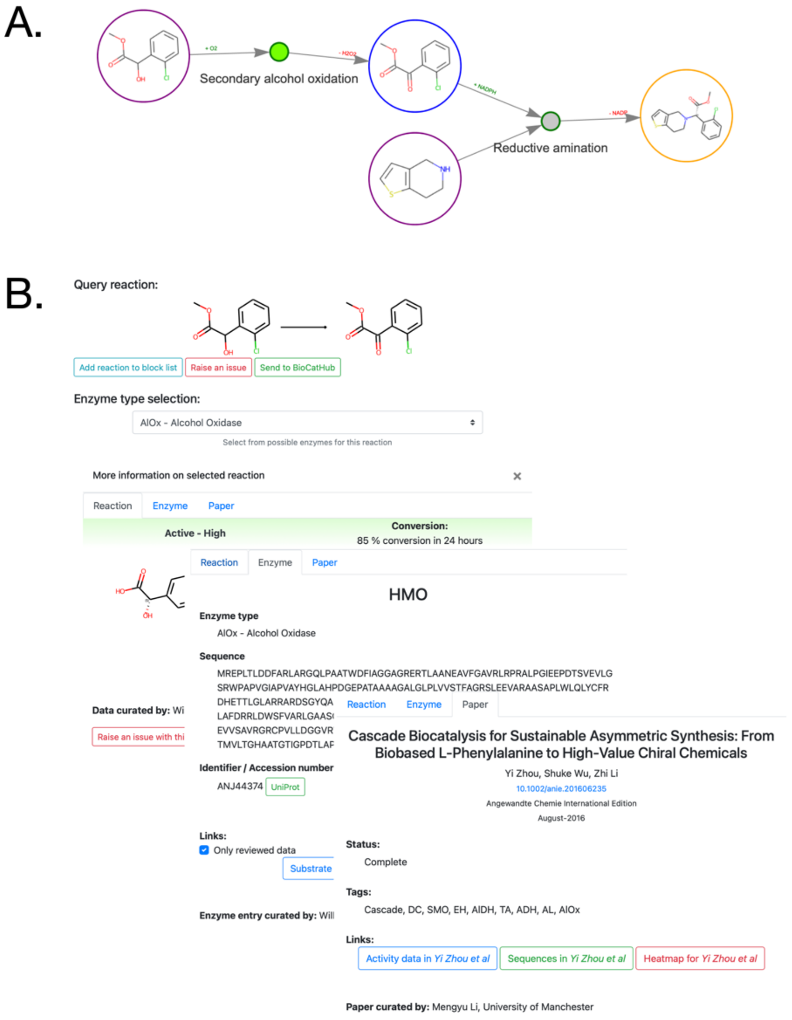

Supplementary Figure 20 – Identification of HMO as a possible enzyme for the secondary alcohol oxidation step. A. The pathway identified through the synthesis planning tools available in RetroBioCat. The green reaction node indicates some similar literature precedent has been identified. **B.** Clicking the green node launches a window displaying information about the similar reaction, the enzyme reported, and the paper this is from.

6.2 Identification of a CAR enzyme for the synthesis of 3-ethylbenzaldehyde using the similarity search tool

The similarity search tool can be used to more finely identify enzymes to carry out a proposed biotransformation. Entering a desired product of 3-ethylbenzaldehyde to be produced through a carboxylic acid reduction reaction by a CAR enzymes yields a number of results (**Supplementary Figure 21 - A**). These can be ranked by similarity score, specific activity, or conversion, yielding several options for the best CAR enzymes to select (**Supplementary Figure 21 – B, C and D**). AncCAR-A, msCAR and tpCAR are identified as the best enzymes for reactions which produce the most similar product. However, other CAR enzymes have shown even better activity with other similar substrates. MAB4714, MIM0040, MAB2962 and sroCAR all shown very high specific activities with similar substrates. Likewise, srCAR, mmCAR or niCAR show the highest conversions with other similar substrates. Ultimately, a human decision would need to be made about which of these are the best enzymes to try in the lab.

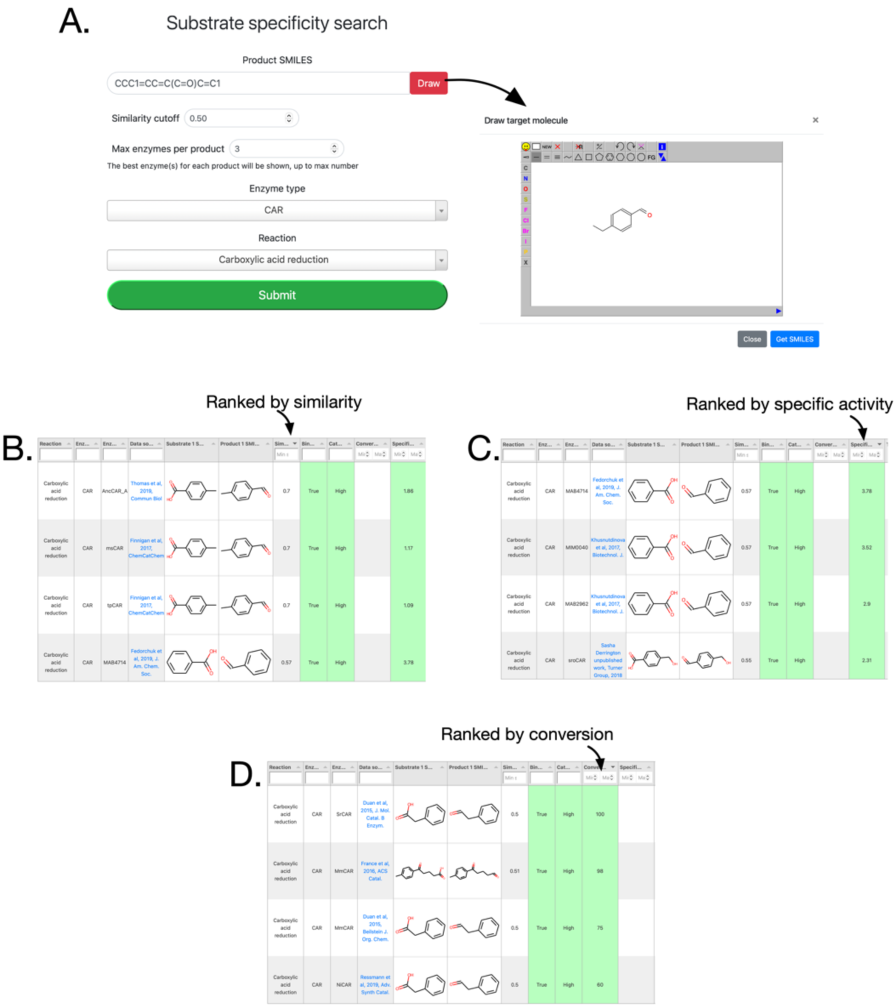

Supplementary Figure 21 – Identification of CAR enzymes for synthesis of 3-ethylbenzaldehyde. A. The entry point for a similarity search. **B.** The results sorted by similarity score, **C.** The results sorted by specific activity, **D.** The results sorted by conversion.

6.3 Identification of an S selective IRED enzyme for the synthesis of (2S)-2-phenylpiperidine using the similarity search tool

Often an enzyme must be selected with the correct enantio-selectivity. Again, the similarity search tool can be useful to identify similar reactions which have shown the same selectivity. For example, to identify an IRED for the synthesis enantio-selective synthesis of (2S)-2-phenylpiperidine through the reduction of the corresponding imine the results of a similarity search can be filtered only for S selective examples (**Supplementary Figure 22**). RIR-Sgf3587 is identified as a good enzyme for this reaction.

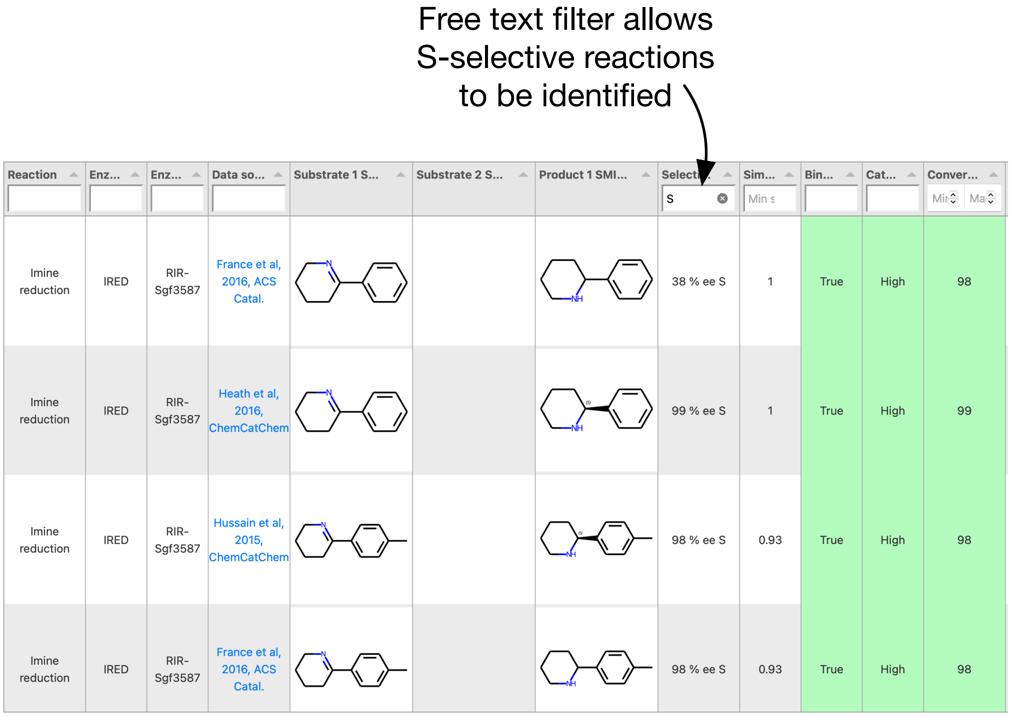

Supplementary Figure 22 – Identification of S-selective reactions for an IRED reaction. The result of a similarity search for the synthesis of (2S)-2-phenylpiperidine by an IRED is shown. Using the free text filter, S selective examples are identified.

7 References

(1) Finnigan, W.; Hepworth, L. J.; Flitsch, S. L.; Turner, N. J. RetroBioCat as a Computer-Aided Synthesis Planning Tool for Biocatalytic Reactions and Cascades. *Nature Catalysis* **2021**, *4* (2), 98–104. https://doi.org/10.1038/s41929-020-00556-z.

(2) Chang, A.; Jeske, L.; Ulbrich, S.; Hofmann, J.; Koblitz, J.; Schomburg, I.; Neumann-Schaal, M.; Jahn, D.; Schomburg, D. BRENDA, the ELIXIR Core Data Resource in 2021: New Developments and Updates. *Nucleic Acids Research* **2021**, *49* (D1), D498–D508. https://doi.org/10.1093/nar/gkaa1025.

(3) Bansal, P.; Morgat, A.; Axelsen, K. B.; Muthukrishnan, V.; Coudert, E.; Aimo, L.; Hyka-Nouspikel, N.; Gasteiger, E.; Kerhornou, A.; Neto, T. B.; Pozzato, M.; Blatter, M.-C.; Ignatchenko, A.; Redaschi, N.; Bridge, A. Rhea, the Reaction Knowledgebase in 2022.

Nucleic Acids Research **2022**, *50* (D1), D693–D700. https://doi.org/10.1093/nar/gkab1016.
(4) Kanehisa, M.; Sato, Y.; Kawashima, M. KEGG Mapping Tools for Uncovering Hidden Features in Biological Data. *Protein Science* **2021**, pro.4172. https://doi.org/10.1002/pro.4172.
(5) The UniProt Consortium; Bateman, A.; Martin, M.-J.; Orchard, S.; Magrane, M.; Agivetova, R.; Ahmad, S.; Alpi, E.; Bowler-Barnett, E. H.; Britto, R.; Bursteinas, B.; Bye-A-Jee, H.; Coetzee, R.; Cukura, A.; Da Silva, A.; Denny, P.; Dogan, T.; Ebenezer, T.; Fan, J.; Castro, L. G.; Garmiri, P.; Georghiou, G.; Gonzales, L.; Hatton-Ellis, E.; Hussein, A.; Ignatchenko, A.; Insana, G.; Ishtiaq, R.; Jokinen, P.; Joshi, V.; Jyothi, D.; Lock, A.; Lopez, R.; Luciani, A.; Luo, J.; Lussi, Y.; MacDougall, A.; Madeira, F.; Mahmoudy, M.; Menchi, M.; Mishra, A.; Moulang, K.; Nightingale, A.; Oliveira, C. S.; Pundir, S.; Qi, G.; Raj, S.; Rice, D.; Lopez, M. R.; Saidi, R.; Sampson, J.; Sawford, T.; Speretta, E.; Turner, E.; Tyagi, N.; Vasudev, P.; Volynkin, V.; Warner, K.; Watkins, X.; Zaru, R.; Zellner, H.; Bridge, A.; Poux, S.; Redaschi, N.; Aimo, L.; Argoud-Puy, G.; Auchincloss, A.; Axelsen, K.; Bansal, P.; Baratin, D.; Blatter, M.-C.; Bolleman, J.; Boutet, E.; Breuza, L.; Casals-Casas, C.; de Castro, E.; Echioukh, K. C.; Coudert, E.; Cuche, B.; Doche, M.; Dornevil, D.; Estreicher, A.; Famiglietti, M. L.; Feuermann, M.; Gasteiger, E.; Gehant, S.; Gerritsen, V.; Gos, A.; Gruaz-Gumowski, N.; Hinz, U.; Hulo, C.; Hyka-Nouspikel, N.; Jungo, F.; Keller, G.; Kerhornou, A.; Lara, V.; Le Mercier, P.; Lieberherr, D.; Lombardot, T.; Martin, X.; Masson, P.; Morgat, A.; Neto, T. B.; Paesano, S.; Pedruzzi, I.; Pilbout, S.; Pourcel, L.; Pozzato, M.; Pruess, M.; Rivoire, C.; Sigrist, C.; Sonesson, K.; Stutz, A.; Sundaram, S.; Tognolli, M.; Verbregue, L.; Wu, C. H.; Arighi, C. N.; Arminski, L.; Chen, C.; Chen, Y.; Garavelli, J. S.; Huang, H.; Laiho, K.; McGarvey, P.; Natale, D. A.; Ross, K.; Vinayaka, C. R.; Wang, Q.; Wang, Y.; Yeh, L.-S.; Zhang, J.; Ruch, P.; Teodoro, D. UniProt: The Universal Protein Knowledgebase in 2021. *Nucleic Acids Research* **2021**, *49* (D1), D480–D489. https://doi.org/10.1093/nar/gkaa1100.
(6) Buchholz, P. C. F.; Vogel, C.; Reusch, W.; Pohl, M.; Rother, D.; Spieß, A. C.; Pleiss, J. BioCatNet: A Database System for the Integration of Enzyme Sequences and Biocatalytic Experiments. *ChemBioChem* **2016**, *17* (21), 2093–2098. https://doi.org/10.1002/cbic.201600462.
(7) Wittig, U.; Rey, M.; Weidemann, A.; Kania, R.; Müller, W. SABIO-RK: An Updated Resource for Manually Curated Biochemical Reaction Kinetics. *Nucleic Acids Research* **2018**, *46* (D1), D656–D660. https://doi.org/10.1093/nar/gkx1065.
(8) Swainston, N.; Baici, A.; Bakker, B. M.; Cornish-Bowden, A.; Fitzpatrick, P. F.; Halling, P.; Leyh, T. S.; O'Donovan, C.; Raushel, F. M.; Reschel, U.; Rohwer, J. M.; Schnell, S.; Schomburg, D.; Tipton, K. F.; Tsai, M.; Westerhoff, H. V.; Wittig, U.; Wohlgemuth, R.; Kettner, C. STRENDA DB: Enabling the Validation and Sharing of Enzyme Kinetics Data. *FEBS J* **2018**, *285* (12), 2193–2204. https://doi.org/10.1111/febs.14427.
(9) Gunera, J.; Kindinger, F.; Li, S.-M.; Kolb, P. PrenDB, a Substrate Prediction Database to Enable Biocatalytic Use of Prenyltransferases. *Journal of Biological Chemistry* **2017**, *292* (10), 4003–4021. https://doi.org/10.1074/jbc.M116.759118.
(10) Drula, E.; Garron, M.-L.; Dogan, S.; Lombard, V.; Henrissat, B.; Terrapon, N. The Carbohydrate-Active Enzyme Database: Functions and Literature. *Nucleic Acids Research* **2022**, *50* (D1), D571–D577. https://doi.org/10.1093/nar/gkab1045.

(11) Savelli, B.; Li, Q.; Webber, M.; Jemmat, A. M.; Robitaille, A.; Zamocky, M.; Mathé, C.; Dunand, C. RedoxiBase: A Database for ROS Homeostasis Regulated Proteins. *Redox Biology* **2019**, *26*, 101247. https://doi.org/10.1016/j.redox.2019.101247.
